# Recent Growth and Expansion of Birch Shrubs Across a Low Arctic Landscape in Continental Canada: Are These Responses More a Consequence of the Severely Declining Caribou Herd than of Climate Warming?

**DOI:** 10.1007/s10021-019-00474-7

**Published:** 2020-01-09

**Authors:** Rhett Andruko, Ryan Danby, Paul Grogan

**Affiliations:** 1 Department of Biology, Queen’s University, Kingston, Ontario K7L 3N6 Canada; 2 School of Environmental Studies, Queen’s University, Kingston, Ontario K7L 3N6 Canada

**Keywords:** birch, deciduous shrub, Arctic tundra, climate change, caribou, herbivory, trampling, groundcover, soil nutrients, soil moisture

## Abstract

The recent widespread expansion of deciduous shrubs across much of the Arctic has been largely attributed to climate warming. This study investigated decadal growth rates of dwarf birch (*Betula glandulosa*) across a low Arctic landscape in the continental interior of Canada. Detailed birch cover (100 m^2^ replicate plots) and individual shrub stature measurement datasets for five representative habitat-types were compared between 2006 and 2016, and evaluated in relation to environmental characteristics. Furthermore, dendrochronologically-based annual growth rates were assessed in relation to the 20-year climate record. Birch height, lateral dimensions, and patch groundcover all increased 20–25% relative to 2006 values, but these increases were similar among the habitat-types. Together, the limited evidence of recent warming at this site, the absence of significant habitat-type growth rate differences, and the lack of correlation between annual climate and stem secondary growth strongly suggest that climate change was not the principal cause. Instead, we propose that release from caribou impacts following the recent severe herd decline may explain the net shrub growth. Individual shrub growth rates were correlated with soil nutrient availability, but the latter was highly variable, suggesting that growth rates are primarily determined by fine-scale rather than habitat-scale spatial heterogeneity in nutrient supply. Together, our results demonstrate that birch growth has been enhanced across a variety of habitat-types in the Daring Lake landscape over the decade since 2006, and suggest that the recent severe caribou herd declines may be at least as significant as climate warming in driving birch shrub expansion in the Canadian central low Arctic.

**Electronic supplementary material:**

The online version of this article (10.1007/s10021-019-00474-7) contains supplementary material, which is available to authorized users.

## Highlights

- Birch growth and cover across diverse habitat-types have increased by about 25% since 2006.
- Recent climate warming trends at this low Arctic continental interior site are limited.
- Release from caribou impacts due to herd decline may explain the enhanced growth.

## Introduction

### Recent Increases in Arctic Deciduous Shrub Growth: Patterns and Implications

The Arctic is currently undergoing many fundamental changes, one of which is an increase in vegetation productivity that has been widely attributed to climate warming (Comiso and Hall 2014; IPCC 2013). “Greening” has been observed in many locations across the low Arctic tundra of North America in the past 30 years, but especially in coastal and near-coastal regions such as northern Alaska, the northwest coast of Canada, and northern Quebec and Labrador (Beck and Goetz 2011; Ju and Masek 2016). By contrast, these satellite-based studies indicate that there has been relatively little, and very patchy, vegetation greening in more inland tundra regions such as the central and eastern Canadian Arctic (that is, the central Northwest Territories to Nunavut) (Ju and Masek 2016; Bonney and others 2018). Either vegetation change has not occurred over the past 30 years in many areas within the interior continental region, or it is slow and patchy across the landscape and often not detectable because of the low spatial resolution (30 m pixels) of the satellite data available for that period. Furthermore, although these remote sensing observations have been largely supported by various ground-truthing studies in near-coastal Arctic regions (Myers-Smith and others 2011), there has been very little corresponding on-the-ground research within the continental interior.

Increases in the groundcover, density, and stature of deciduous shrub species (*Betula* spp., *Alnus* spp., *Salix* spp.) are the primary explanation for the recent trends in Arctic land surface ‘greening’ (Myers-Smith and others 2011). Given that these species are often the physically dominant vegetation over much of the low Arctic, their increased growth could result in a number of important feedbacks, both within the ecosystem itself and to the climate as a whole. First, tall deciduous shrub canopies accumulate snow that insulates their underlying soils overwinter, significantly restricting heat loss and winter soil temperature minima that can promote permafrost thaw (Tape and others 2006; Myers-Smith and Hik 2013). Enhanced microbial decomposition of Arctic soil organic matter as a result of warmer winter soils and permafrost thaw could release large quantities of greenhouse gases, including CO_2_ and CH_4_ (Schuur and others 2015). Second, areal expansion and vertical extension of deciduous shrub canopies can alter the overall energy balance of tundra terrestrial ecosystems by reducing land surface albedo (reflectance of incoming solar radiation), resulting in earlier snowmelt and warmer local air masses that would amplify the effects of climate change in the Arctic (Chapin and others 2005). Third, deciduous shrub canopies strongly influence the overall composition and structure of vegetation communities in Arctic tundra. Deciduous shrubs shading of understory communities can significantly reduce plant species richness (Pajunen and others 2011), but their extensive height and woody structure may also protect the understory from large herbivore impacts, thereby enhancing richness (Bråthen and Lortie 2016). They also enhance soil nitrogen availability through their effects on soil microclimate and their relatively large litter production (Buckeridge and others 2010; DeMarco and others 2011; Vankoughnett and Grogan 2016), promoting their own growth and inhibiting neighboring plant species such as evergreens that are not as well adapted to elevated soil fertility (Zamin and others 2014).

### Factors Affecting Net Deciduous Shrub Growth in Arctic Tundra

**Figure 1:**
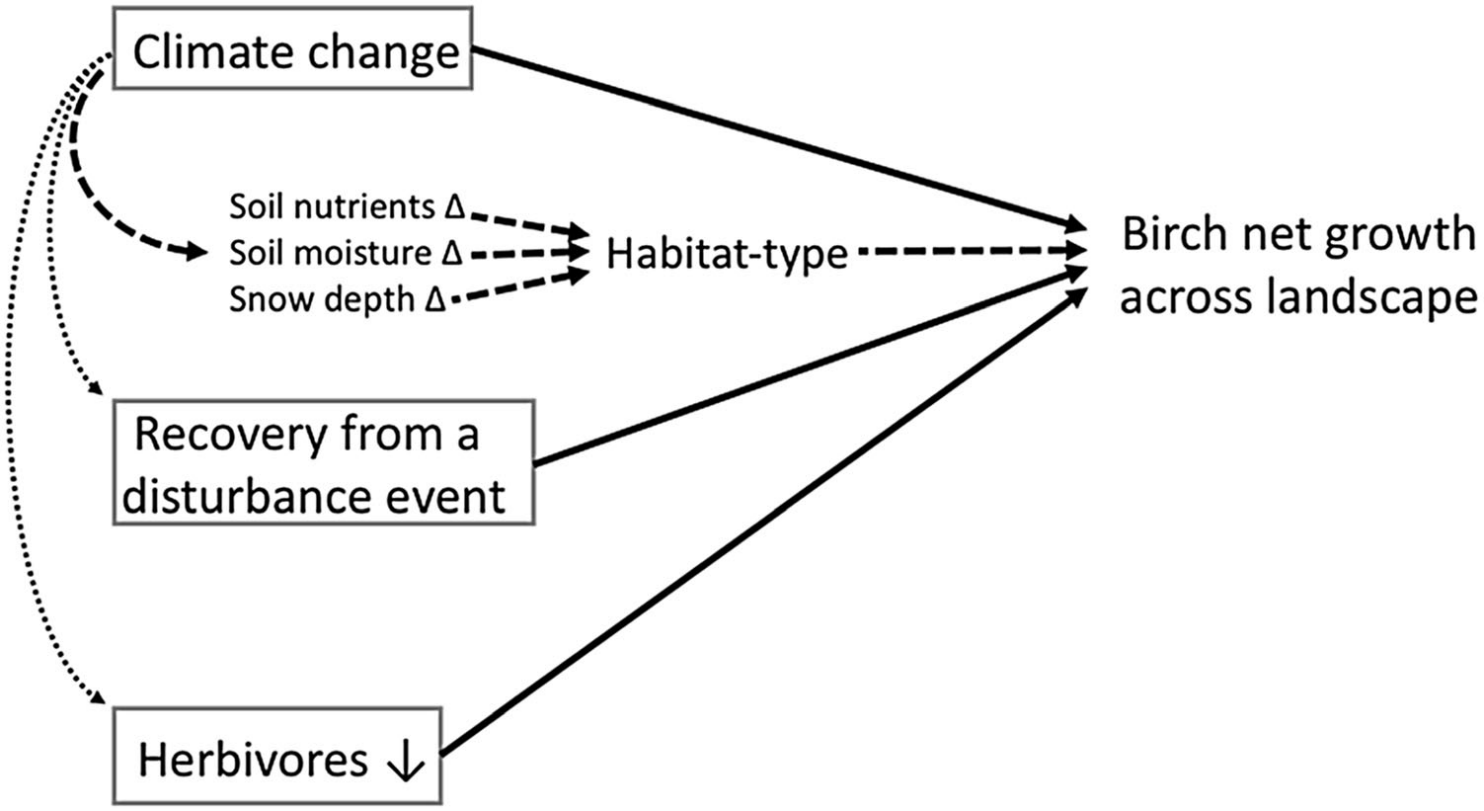
A conceptual framework of the three principal factors (gray boxes) that can directly determine landscape-scale patterns of net decadal growth in tundra deciduous shrubs (such as birch). Climate change can indirectly affect deciduous shrub growth by altering environmental features of habitat-type (thick dashed black lines; Δ indicates change), and can also exert significant interaction effects on the other two principal factors (thin dotted lines; see text for details).

Climate change, reduced herbivore populations (for example, of voles, hares, and caribou), and recovery from ecosystem disturbances (for example, fire, permafrost degradation, human disturbance) are the three main factors that can drive phases of net growth (that is, sustained biomass increases over multiple years) in established deciduous shrubs (Myers-Smith and others 2011). We developed a conceptual framework that includes these principal factors, but also specifically incorporates the potential mediating influence of habitat-type, as well as the main interactions between climate change and the other main factors (Figure 1). For example, climate change could directly affect shrub growth through warming-enhanced photosynthesis, and/or it could alter soil fertility, soil moisture or snow depth, indirectly affecting shrub growth. Furthermore, the potential effects of climate change on these environmental components may vary in magnitude, and even direction, depending on the distinctive characteristics of each particular deciduous shrub habitat-type (Figure 1). In terms of interactions, climate change could promote disturbance events (for example, permafrost thaw) that may subsequently stimulate shrub growth. Finally, climate change could reduce or enhance herbivore population sizes, resulting in a decrease or increase, respectively, in their top-down regulatory impacts (that is, direct consumption of plant tissue, but also trampling damage in the case of large mammals such as caribou) on shrub net growth (Figure 1).

Of the three principal factors, ongoing regional climate warming is generally assumed to be the main factor driving the satellite, landscape repeat photography and dendrochronologically based reports of enhanced deciduous shrub growth across much of the low Arctic (for example, Ju and Masek 2016; Tape and others 2012; Tremblay and others 2012; Ropars and others 2017). Furthermore, meta-analyses of standardized experimental warming treatments across the Arctic have provided rigorous evidence that deciduous shrub growth can be significantly enhanced by elevated summer temperatures (Walker and others 2006; Elmendorf and others 2012a).

However, the factors that affect the growth rates of individual shrubs in response to warming remain poorly understood, and large-scale meta-analyses of recent changes in tundra shrub communities indicate considerable spatial heterogeneity in growth rates that is attributed not just to variability in temperature trends, but also to soil moisture differences (Elmendorf and others 2012b). Low Arctic tundra landscapes are highly diverse, with distinct topographically-determined gradients of vegetation ranging from the sparse, low plant cover on the tops of exposed dry ridges to the thick, tall shrub canopies within wind-protected watercourse channels or on valley stream slopes and floodplains. Snow accumulation and subsequent meltwater inputs and flows in spring, wind exposure, and the soil’s depth and moisture-holding characteristics also vary substantially across these gradients (Giblin and others 1991) resulting in strong soil fertility differences between adjacent tundra habitat-types (Björk and others 2007; Chu and Grogan 2010). Therefore, the particular habitat-type associated with topographic location may be an important underlying factor determining the growth responses of individual shrubs to warming, and hence of the overall landscape-scale patterns of shrub growth responses.

Indeed, most previous repeat photography and dendrochronological studies have generally concluded that tundra deciduous shrub growth responses to recent warming differ according to habitat-type (Tape and others 2006; Naito and Cairns 2011; Tape and others 2012; Tremblay and others 2012; Ropars and Boudreau 2012; Blok and others 2011; Ropars and others 2015; Cameron and Lantz 2016; Young and others 2016; Ackerman and others 2017). For example, in response to recent warming, alder (*Alnus*) shrub patches in relatively nutrient-rich habitat-types (for example, in floodplains and stream corridors) have tended to exhibit net growth and expansion, whereas those in more nutrient-poor habitat-types (for example, along ridges) have tended to remain stable (Tape and others 2012). However, the mediating influence of habitat-type on deciduous shrub growth rates has only been explicitly demonstrated in a few studies (cited above), all of which were in tundra regions where significant recent climate warming *and* greening are occurring—such as northern Alaska, northern Quebec, and the northern mainland coast of Canada—while none were located in continental interior regions such as the central Canadian Arctic.

**Figure 2:**
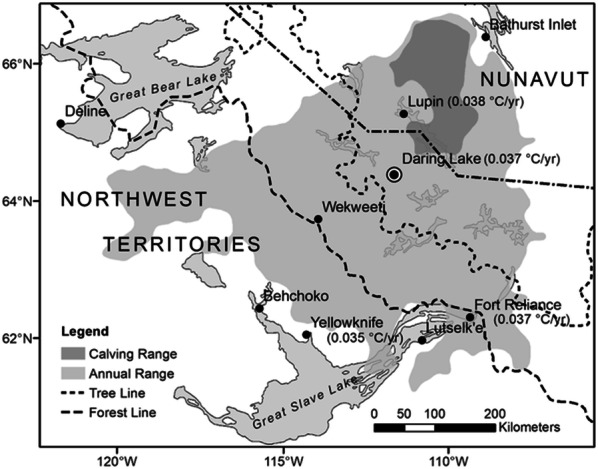
The location of the Daring Lake study area in relationship to the Bathurst caribou herd’s annual range and calving area, as inferred from GPS collar locations obtained from 1996–2015 (Government of Northwest Territories 2019). The three closest Environment and Climate Change Canada (ECCC) meteorological stations are located at Yellowknife, Lupin, and Fort Reliance. Data beside each station name, as well as for Daring Lake, indicate the annual linear increase in temperature since 1950, as interpolated from the CRU v.4.3 database (Harris and others 2014). Strongly positive correlations exist between instrumental temperature records at each station and the CRU data (see Methods text).

Our study focused specifically on the deciduous shrub dwarf birch (*Betula glandulosa* Michx.—hereafter referred to as “birch”) as it is the most dominant component of erect shrub canopies in the low Arctic’s continental interior (Porsild and Cody 1980; CAVM 2003). Birch shrubs are particularly interesting in terms of deciduous shrub net growth dynamics because they have the ability to quickly allocate resources to lateral shoots under favorable conditions, allowing them to dominate several tundra communities when and where soil fertility is enhanced (Bret-Harte and others 2001). We studied birch growth rates in a variety of its habitat-types at the Daring Lake site which is located in the central Canadian low Arctic and is also close to the center of the Bathurst caribou herd’s annual migratory range (Figure 2). Our research addressed the following specific questions:

1. Have there been net increases in birch shrub groundcover and stature (that is, height, lateral dimensions, branch length) across the Daring Lake landscape from 2006–2016, and if so, did the increases differ among habitat-types?
2. Do dendrochronologically-based birch stem growth rates correlate with either interannual variations or longer-term climate trends at Daring Lake?
3. Can habitat-type differences in environmental factors such as soil moisture and nutrient availabilities be used to predict growth rates of individual birch shrubs?

## Methods

### Study Site

This study was performed near the Tundra Ecosystem Research Station (TERS) at Daring Lake, Northwest Territories (64° 52′ N, 111° 33′ W) (Figure 2). A meteorological station has been operated by Kokelj and others (Water Management and Monitoring Division of the Department of Environment and Natural Resources, Government of Northwest Territories) at TERS since 1996. Measured daily mean air temperatures range from about 14°C in July to about − 29°C in January, and the landscape is typically covered in snow from October to May. Monthly average temperature data from the station exhibit strong, positive correlations with records from the nearest Environment and Climate Change Canada (ECCC) meteorological stations at Yellowknife (*r* = 0.990, *p* < 0.001), Fort Reliance (*r* = 0.993, *p* < 0.001), and Lupin (*r* = 0.999, *p* < 0.001; locations indicated in Figure 2) where much longer instrumental records are available (1943–2018, 1949–2018, and 1959–2018, respectively). Based on these strong correlations, there is no reason to expect that the area around Daring Lake would have exhibited different trends prior to the instrumental data collection that began there in 1996. A variety of herbivores occur at the Daring Lake site including red-backed voles (*Myodes rutilus*), ptarmigan (*Lagopus spp.*), arctic hares (*Lepus arcticus*), caribou (*Rangifer tarandus*), and very occasional moose (*Alces alces*).

Five distinct habitat-types in which birch occurs were identified in the terrain around Daring Lake (Appendix A.1) based on various factors known to be involved in structuring tundra environments along topographic gradients including snow accumulation patterns, moisture and drainage patterns, wind exposure, and vegetation structure. Two completely separate areas (at least 200 m apart) of each habitat-type were selected, and a 10 × 10 m plot was established in each in 2006 (See photos in Appendix A.2). Four representatives of typical birch plants within each plot were chosen as ‘focal’ shrubs.

### Birch Shrub Cover and Stature Measurements

Birch cover in each 100 m^2^ plot was carefully mapped by hand at a scale of 1:50 in 2006 and 2016 by subdividing the plot into 2 × 2 m^2^, and sketching the extent of birch areal coverage within each square (using direct measurements of shrub sizes to scale sketches appropriately). Since the data were collected by different (single) researchers in 2006 versus 2016, the precision of the sketch maps produced from the two sampling years was tested by comparing the direct measurements of the four focal shrubs in each plot (described below) to the map-inferred areal extent of each of these shrubs for each of the sampling years. We found no statistically significant differences in bias between the two researchers’ data sets, indicating that the temporal comparison of cover change is robust (see Appendix B for details of comparison). Cover maps were scanned (~ 4700 × 4700 pixels), and areas of shrub cover filled in with black (Paintbrush 2.1.2) before resolving the pixels to either black or white and determining the % of the former (using ImageJ 1.50i).

Morphological measurements of each focal birch were made in both 2006 and 2016 as follows: (1) maximum height; (2) canopy areal coverage (that is, shrub horizontal extent as determined by length and orthogonal width dimensions in the ground surface plane, using *x* and *y* directions relative to the plot borders); and (3) number and length of primary shoots (defined as those emanating from the central root collar). We calculated ‘lateral dimensions average’ as the mean of the length and orthogonal width measurements, and ‘total primary shoot length’ as the summed lengths of all primary shoots on a shrub.

### Birch Stem Ring-Width Dendrochronology Measurements

Utilizing accepted dendrochronological methodology (that is, consistent with that used in similar studies such as Tape and others 2012, and Ropars and Boudreau 2012), stem sections were obtained from the base of the largest central shoot (as close to the ground as possible) of 25 birch shrubs that were located adjacent to each 100 m^2^ plot. These samples were obtained from distinct individual plants several meters apart that were more than 4 m from the 100 m^2^ long-term monitoring plots (to avoid disturbance), and in areas visually matching the selected habitat-type. Larger shrubs with a dominant main stem were sampled preferentially in order to maximize the temporal extent and quality of ring-width series. Stem samples were soaked in water for 18–24 h, after which thin cross sections were obtained with a sliding microtome and stained with toluidine blue to improve ring visibility (Appendix C). Ring-width series along two separate radii for each stem were obtained with MeasureJ2X software (VoorTech Consulting), using a Velmex sliding measurement stage.

Ring-width series were visually cross-dated within habitat-types using index years of particularly high and low growth as references. Cross-dating was statistically validated using the software COFECHA (Tree-Ring Lab, Colombia University). Each series was individually detrended with the program ARSTAN (Tree-Ring Lab, Colombia University) using either a negative-exponential curve or linear regression line (Dearborn and Danby 2018). Ring-width series from 7–9 shrubs in each habitat-type were selected as the most confidently cross-dated, and then averaged to yield master chronologies for each habitat-type. Series that were confidently cross-dated, but that were not well correlated with other series from the same habitat-type were not included in the master chronology for each habitat-type.

### Habitat-type Environmental Characteristics

Soil moisture was measured on three evenly separated dates between early-July and mid-August using a handheld meter (CS616, Campbell Scientific) with the probes inserted vertically to their full extent (that is, integrating from 0–12 cm soil depth), or where soils were thinner, at an angle to ensure the probes were completely immersed. Data are reported here as means of four measurements taken at random locations within about 20 cm of the root collar of each focal shrub.

Soil nutrient availability (NH_4_^+^, NO_3_^−^, and PO_4_^−^) was measured in each plot using Ion Exchange Membranes (IEMs) using a protocol adapted from Giblin and others, 1994—(See Appendix D for full details). Briefly, 5 × 5 cm sheets of the cation and anion material (GE Power and Water: CR67HMR and AR204SZRA) were charged in 0.5 M HCl and 0.5 M NaHCO_3_, respectively. Two IEMs of each ion type were inserted about 2–3 cm below the soil surface at random locations within about 20 cm of the root collar of each focal shrub, and left in situ for 32 days (July 14–August 15), after which they were removed, rinsed with distilled water, and refrigerated. IEMs were eluted in a 2 M NaCl + 0.1 M HCl solution (Appendix D), and eluent nutrient concentrations were determined colorimetrically using automated flow analyses (Bran-Leubbe AutoAnalyzer III, Germany). Recent replicated membrane incubation tests in our laboratory using a range of standard solution concentrations for each nutrient and the elution procedure described above, indicated that these membranes can detect NH_4_–N fluxes above 0.25 μg/cm^2^, and NO_3_–N and PO_4_–P fluxes above 0.05 μg/cm^2^ with an accuracy of ± 20% (Gu and Grogan, unpublished data). These minimum detection limits for ammonium and phosphate were well below the actual amounts accumulated on the majority of field-incubated IEMs, but not for nitrate which hence is excluded from the results reported below.

Active layer depth (ALD) at each focal shrub was estimated by measuring ALD with a metal probe at the nearest plot corner in late August. Snow depth (minimum peak winter value) was estimated at each shrub using either: (1) previous field measurements (Tussock/Sedge habitat-types); (2) photographs and previous observations where appropriate (Esker Plains habitat-type); or (3) by directly measuring the topographic depression at each shrub relative to a nearby raised topographic feature (Watercourse/Snowpack habitat-types) (Appendix E).

Occasional malfunctions on the main TERS climate station resulted in some missing data for snowmelt dates since 2004, and for 2012 air temperatures, and so complementary data were obtained from additional Daring Lake climate stations (Lafleur and Humphreys, unpublished data).

### Statistical Analyses

Shrub stature and environmental variables at each focal shrub were assumed to be independent of each other and so were treated as replicate units (*n* = 8) for computing habitat-type means and standard errors. Significant changes over time in shrub stature variables within each habitat-type were identified using one-sample t tests that compared the decadal changes for individual shrubs against a null mean of 0. Differences in net shrub growth and in environmental variables among habitat-types were investigated with nested ANOVAs, using a mixed-effect model with habitat-type as a fixed effect and plot as a random effect with focal shrub nested within plot, followed by Tukey means comparison. These analyses were performed in *R* using the package *lme*4 (Bates and others 2015). Correlations between variables were computed using Pearson’s product-moment linear correlation coefficient. Both the ammonium and phosphate flux data had strongly right-skewed distributions, and the ammonium data, in particular, had many zero (that is, below detection limit) values (15 out of 40). However, natural log transformations of either data set did not substantially alter the overall patterns of statistical results obtained with untransformed data, and so the latter were used in all analyses.

Pearson’s product-moment linear correlations were used to relate the master ring-width chronologies for each habitat-type to various annual meteorological variables. To compare variability in shrub growth among habitat-types, we calculated the standard deviation in annual ring-width for each detrended series and then calculated an average standard deviation for each habitat-type.

All climate analyses reported are based on either monthly or annual values for each year from 1996–2016, and monthly anomalies were calculated as the difference between the monthly variable and its 20-year average. Climate trends over time were tested by computing Pearson’s product-moment linear correlation coefficient.

## Results

### Birch Plot Cover and Focal Shrub Stature

**Figure 3:**
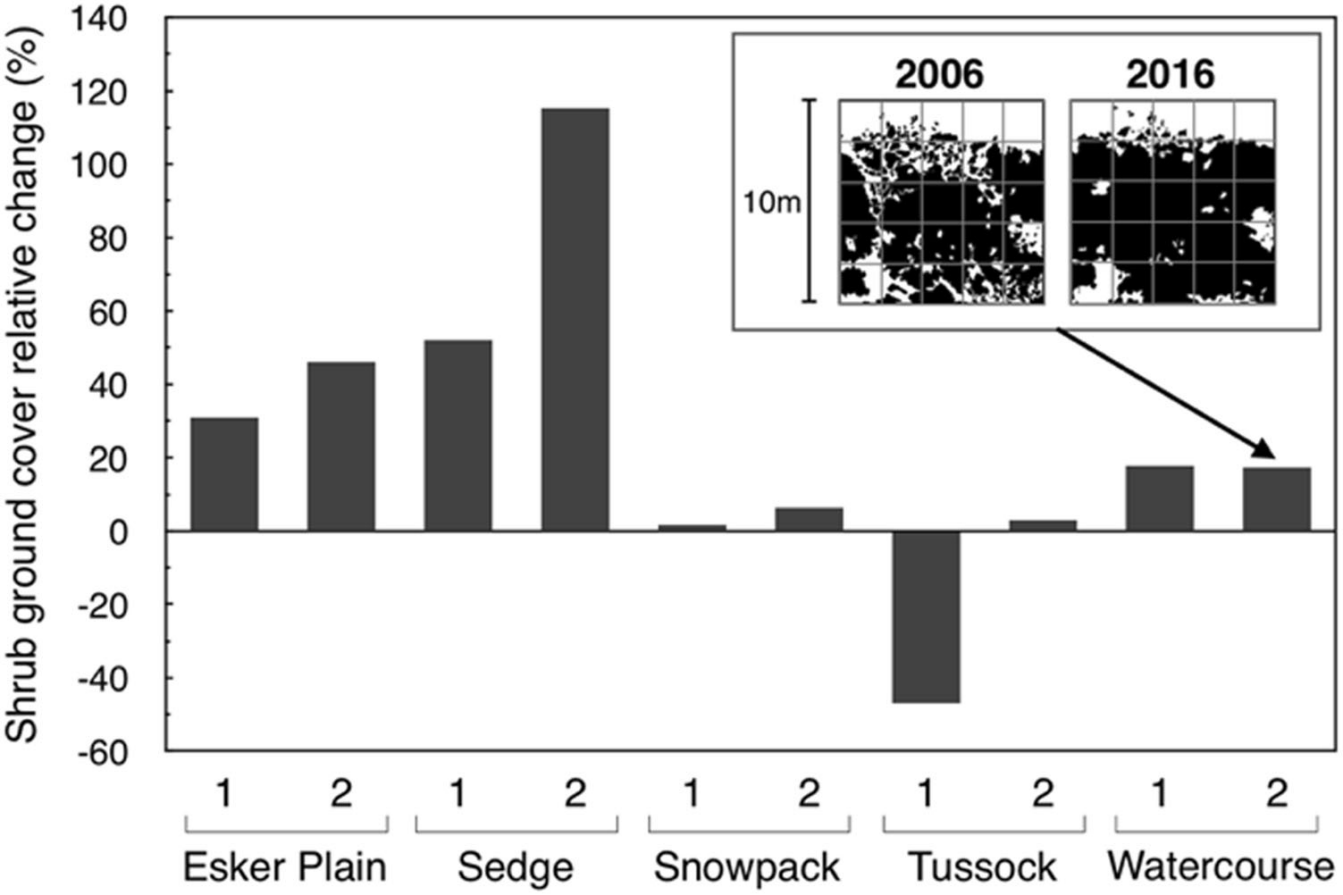
Birch shrub cover relative change from 2006 to 2016 (that is, change in cover relative to initial 2006 cover) in each of the duplicate long-term monitoring 100 m^2^ plots for each of the five major habitat-types in the Daring Lake low arctic tundra landscape. The inset diagram illustrates an example (for the Watercourse habitat-type second plot) of the actual cover data in the two sampling years that were used to compute the relative cover change.

Birch cover increased in 9 of the 10 plots between 2006 and 2016 (Table 1), and there was a significant absolute increase in average total groundcover across the landscape from 28 to 32% (Paired *t* test: *t*_1,9_ = 2.73, *P* < 0.023). This small overall increase masks strong spatial heterogeneity in growth responses among the duplicate plots and among habitat-types. Furthermore, the extent of initial (2006) cover differed greatly among habitat-types and plots (Table 1). Accordingly, the relative cover changes (that is, % cover change divided by initial % cover) were large and highly variable compared to the absolute increases, with the mean birch cover increase relative to 2006 cover averaging 24% across all plots, and ranging from 115 to − 47% (Figure 3, Table 1). Finally, although birch cover was generally most extensive in the Watercourse and Snowpack habitat-types, the largest relative increases were in the Esker Plain and Sedge habitat-types, which are located in the driest and wettest topographic parts of the landscape, respectively (Figure 3, Appendix A.1).

**Table 1:** Birch Groundcover (%) in Each of the Duplicate 100 m^2^ Plots Within the Five Major Shrub Habitat-Types in the Daring Lake Landscape in 2006 and 2016, and Its Absolute and Relative (that is, % Cover Change Divided by Initial % Cover to Account for Differences in Initial 2006 Cover) Increases Over that Decade

Habitat-type	Duplicate plot#	Birch groundcover (%)		Absolute change in % cover	Relative change in cover (%)
		2006	2016		
Esker plain	1	24	32	8	31
	2	17	25	8	46
Sedge	1	5	8	3	52
	2	9	20	11	115
Snowpack	1	46	47	1	2
	2	54	58	3	6
Tussock	1	13	7	− 6	− 47
	2	13	13	1	3
Watercourse	1	34	40	6	18
	2	62	72	11	17
Overall mean		28 (6.4)	32 (7.0)	4	24

Parentheses indicate standard errors.

**Figure 4:**
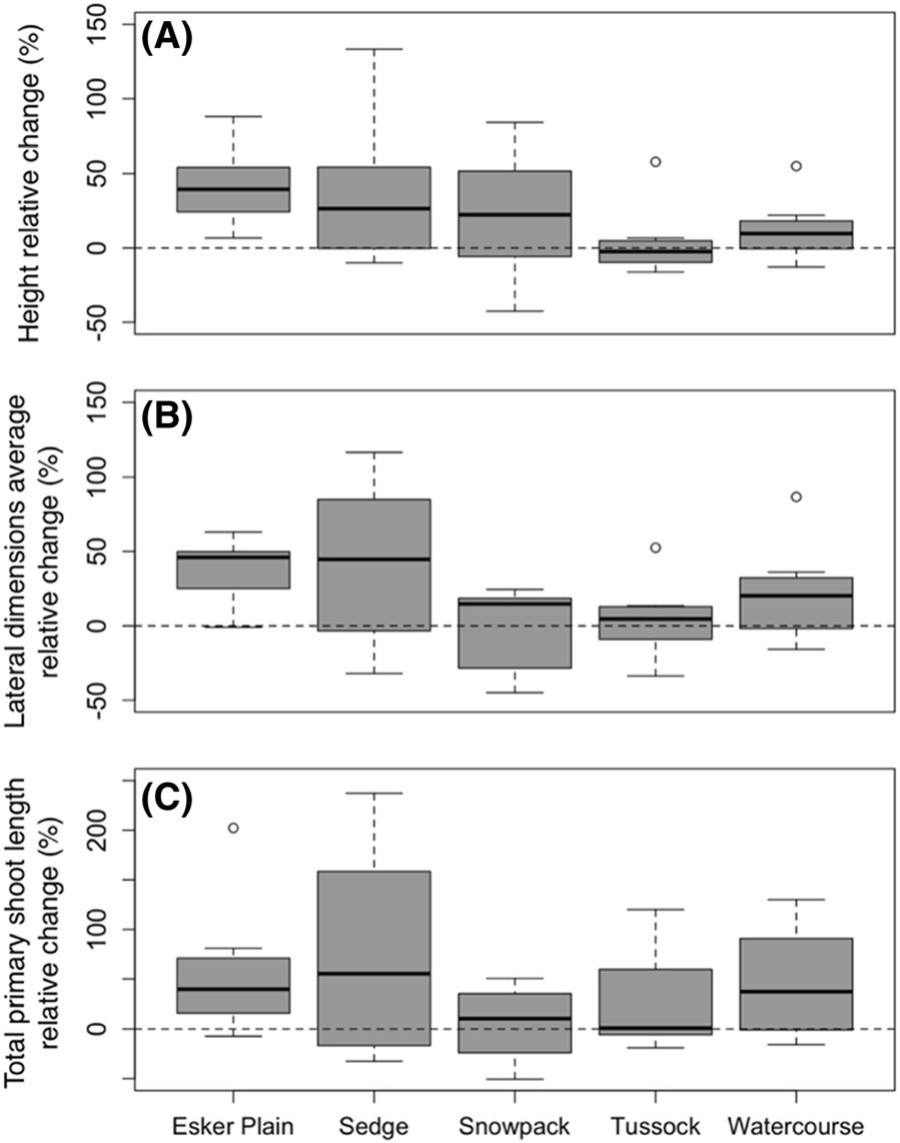
Mean relative changes in birch shrub height **A**, lateral dimensions average **B** and total primary shoot length **C** for the four focal shrubs in the duplicate plots of each habitat-type over the 2006–2016 decade (that is, change is computed relative to initial 2006 values; *n* = 8). Note the scale differences in the Y axes. Bolded lines indicate the median; boxes and whiskers represent the 25th and 75th, and 0th and 100th percentiles, respectively (excluding outliers). White dots indicate outliers, which are defined as values more than 1.5 times the interquartile range either below the 25th or above the 75th quartiles.

The detailed morphological measurements of the individual focal shrubs in each plot also indicated substantially enhanced growth. Mean height in 2016 relative to 2006 increased by 23% on average (*t* = 4.20, *P* < 0.001), and these increases varied from 3 to 41% among the habitat-types (Figure 4, Table 2). However, there was strong variability in height change among individual shrubs within each habitat-type (see Figure 4 data distributions; and Table 2 standard errors of the relative increases—which are based on paired measures of the same shrub over time and were often large enough to exceed half the mean relative change), resulting in no overall statistically significant differences among habitat-types (or plots). Likewise, the lateral dimensions average (that is, the average of canopy areal length and width) increased on average by 25% relative to 2006 values (*t* = 3.06, *P* < 0.004), and these increases varied from − 1 to 60% among the habitat-types (Table 2), but again strong variability among individual shrubs precluded statistically significant differences among habitat-types (or plots). Furthermore, and consistently matching the growth responses reported above, mean total primary shoot length increased by 42% relative to 2006 values (*t* = 3.90, *P* < 0.001), and these increases varied from 5 to 75% among the habitat-types (Table 2), but once again strong variability among individual shrubs (Figure 4, Table 2) precluded statistically significant differences among habitat-types (or plots).

**Table 2:** Mean Height, Lateral Dimensions Average, and Total Primary Shoot Length for the Eight Focal Birch Shrubs in the Duplicate Plots of the Five Major Habitat types Within the Daring Lake Landscape in 2006 and 2016, and Their Absolute and Relative (that is, Accounting for Differences in Initial 2006 Values) Increases Over that Decade

Habitat type	Height				Lateral dimensions average				Total primary shoot length			
	2006 (cm)	2016 (cm)	Absolute increase (cm)	Relative increase (%)	2006 (cm)	2016 (cm)	Absolute increase (cm)	Relative increase (%)	2006 (cm)	2016 (cm)	Absolute increase (cm)	Relative increase (%)
Esker plain	30	42	12	41	75	97	22	38	340	450	110	56
	(4.4)	(5.5)	(2.5)	(9.0)	(10)	(10)	(4.9)	(7.7)	(86)	(74)	(40)	(23)
Sedge	31	42	11	36	33	48	15	60	120	170	57	75
	(4.3)	(7.1)	(4.1)	(17)	(6.4)	(12)	(8.2)	(32)	(25)	(28)	(31)	(37)
Snowpack	43	50	7	22	82	84	2.5	− 1.3	550	660	100	5
	(5.7)	(7.2)	(4.7)	(15)	(14)	(19)	(7.0)	(10)	(160)	(210)	(79)	(14)
Tussock	28	28	0	3.4	32	33	1.5	4.5	140	150	7.2	26
	(3.0)	(2.6)	(1.8)	(8.3)	(5.6)	(7.2)	(3.5)	(8.8)	(50)	(45)	(11)	(17)
Watercourse	44	48	3.9	12	51	56	5.3	22	170	200	34	46
	(14)	(15)	(2.0)	(7.3)	(15)	(15)	(5.2)	(11)	(51)	(51)	(16)	(19)
Overall mean				23				25				42

(*n* = 8; parentheses indicate standard errors).

**Figure 5:**
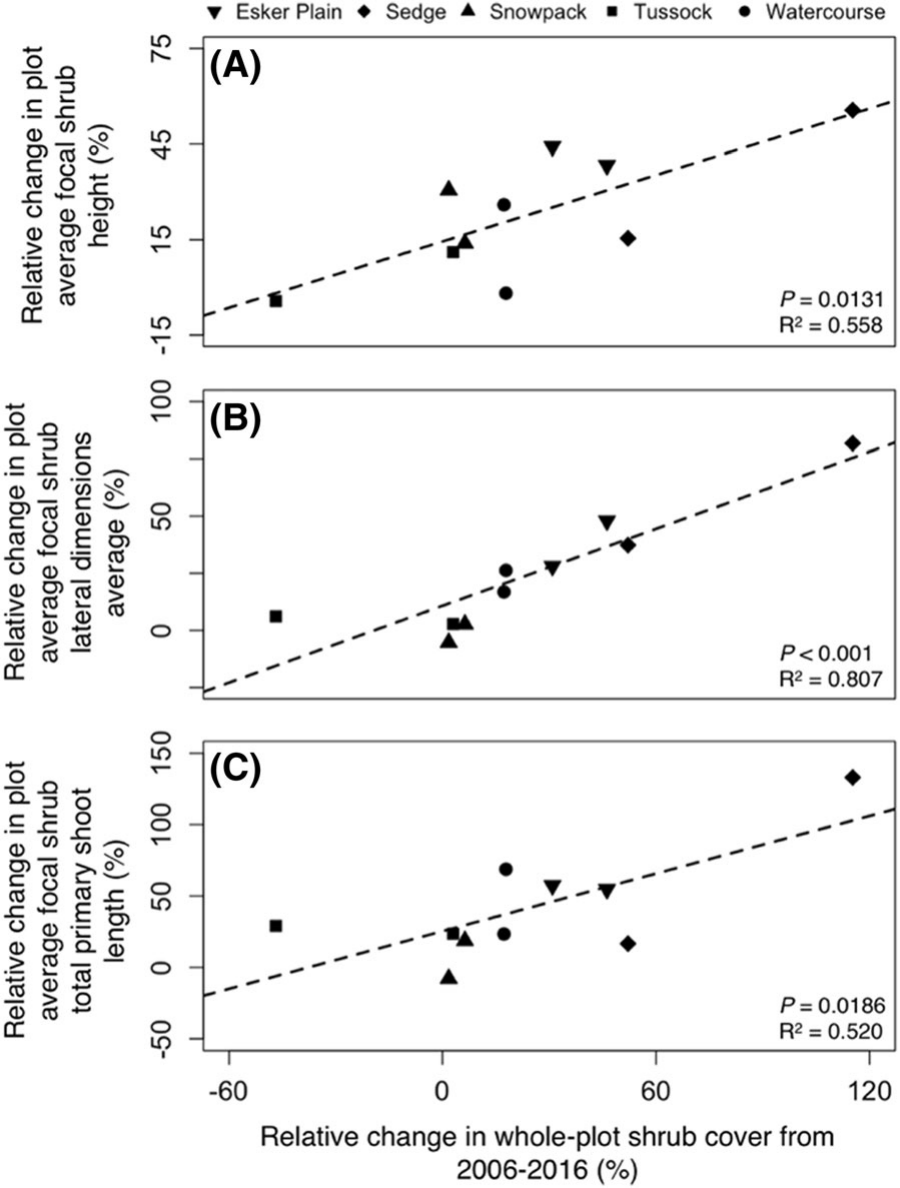
Relative changes in birch cover in each of the ten 100 m^2^ plots over the 2006–2016 decade in relation to the relative change over that same period in plot-averaged focal birch shrub (*n* = 4) height **A**, lateral dimensions average **B**, and total primary shoot length **C**. Note the scale differences in the Y axes.

Overall, these plot cover and focal shrub datasets, representing two distinct metrics at two different spatial scales, were remarkably consistent in indicating not just statistically significant but also similar magnitude increases in birch relative growth over the decade since 2006. Further evidence of this profound consistency is the significant and strong correlations between the relative cover changes in each plot over the decade and the plot averages of relative change in each of the focal shrub stature metrics (Figure 5; *R*^2^ values indicate 50–80% of the total variation explained).

### Birch Shrub Dendrochronology

**Figure 6:**
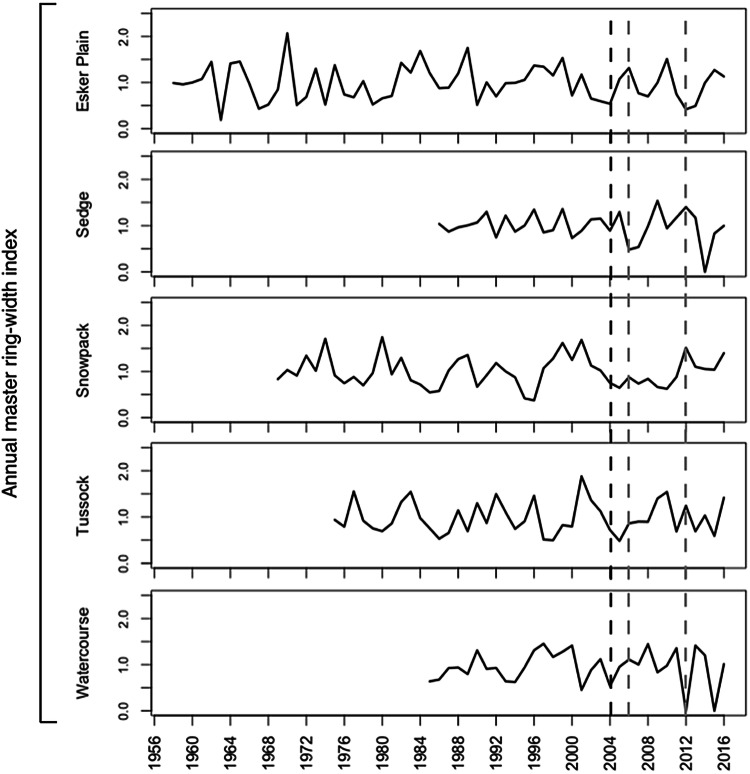
Master annual birch stem ring-width index chronologies for each habitat-type. Data are based on those 7–9 shrubs that were most confidently cross-dated (see Methods) from the 25 oldest/largest shrubs sampled adjacent to the outside perimeter of the duplicate long-term monitoring plots of each habitat-type. The 2004 (black), 2006 and 2012 (both gray) dashed lines highlight relatively cool, and warm years, respectively (see climate data Figure 8).

The master ring-width index chronologies for the five habitat-types spanned from 30 to 58 years (Figure 6). Although we sampled 25 birch shrubs per habitat-type, only 7–9 of them could be confidently cross-dated because of inconsistencies in growth patterns between individual shrubs within plots. The resulting master chronologies for each habitat-type were highly variable, with no visible patterns of shared synchronous peaks or troughs (Figure 6; see for example habitat-type variation for 2004 which was a relatively cool year, and 2006 and 2012, both of which were relatively warm years; full climate data are reported below). Annual ring-width indices were not significantly correlated between any two habitat-type chronologies, and there were no significant temporal trends in any habitat-type chronology (Figure 6). Finally, we tested for relationships between each of 45 different climate variables (for example, monthly averages of daily mean and maximum air temperatures, growing degree days, and so on) and each habitat-type chronology (225 tests in total), and found no more statistically significant correlations than would be expected by chance.

### Soil Environmental Characteristics of the Habitat-types

**Figure 7:**
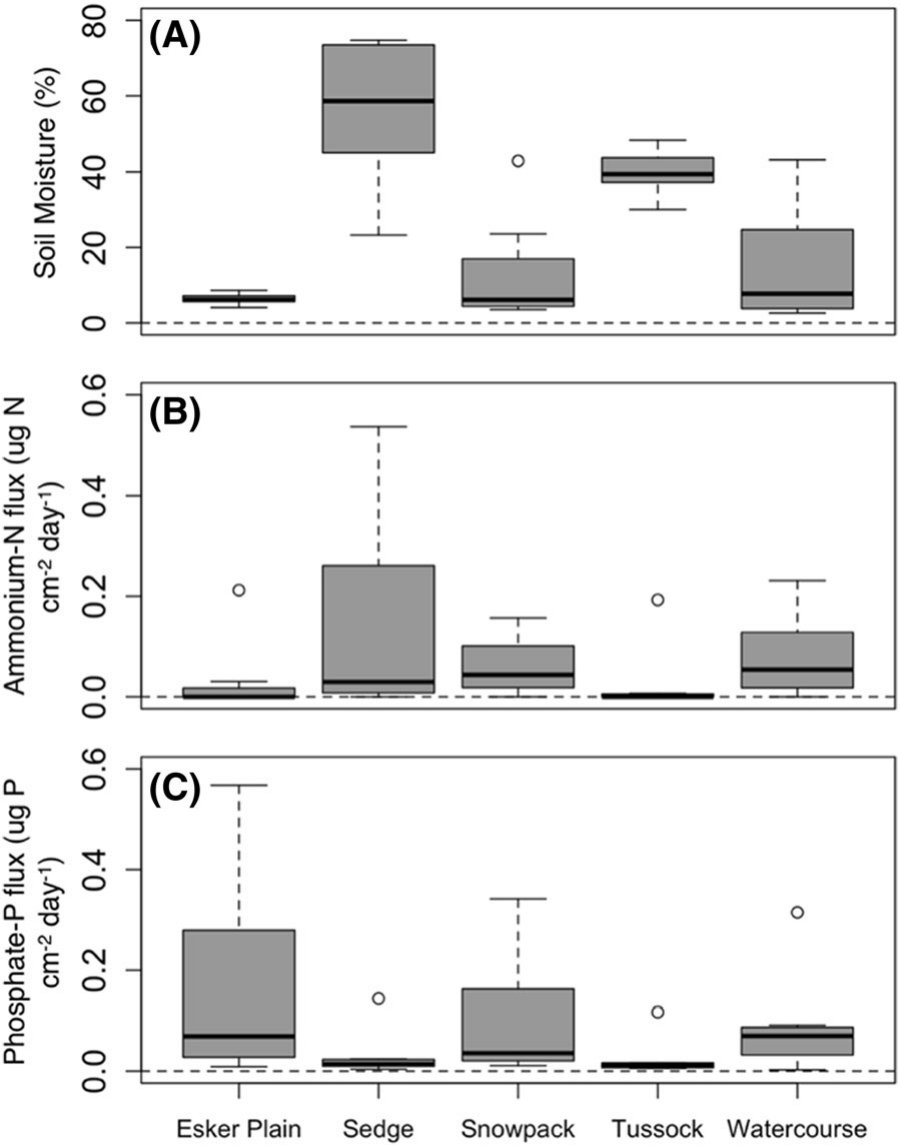
Mean soil moisture **A**, and ammonium and phosphate fluxes **B**, **C** beneath the focal birch shrubs in the duplicate plots of the five major birch habitat-types in the Daring Lake landscape in July–August (*n* = 8; see Figure 4 for explanation of boxplot format). Soil moisture (volumetric water content %) was measured at the base of each shrub (to 12 cm soil depth) three times from early-July to mid-August and averaged for the data presented here. Soil nutrient fluxes were measured by incubating ion exchange membranes from mid-July to mid-August (see Methods for details).

Mean summer soil moisture at the base of the focal birch shrubs was significantly wetter in the Sedge and Tussock habitat-types (*F*_(4,5)_ = 8.42, *P* = 0.019; Figure 7), both of which are located in low-lying areas (Appendix A.1). By contrast, the Snowpack and Watercourse habitat-type soils were on average more than three times drier, and the Esker Plain more than seven times drier (Figure 7).

Mid-summer ammonium and phosphate fluxes in the focal shrub soils were highly variable and did not differ statistically among habitat-types (Figure 7). Nevertheless, at the scale of the individual plants, the relative increases in birch height, lateral dimensions average, and total primary shoot length over the decade from 2006 were significantly or nearly significantly (*P* < 0.081 for primary shoot length) correlated with the soil ammonium fluxes beneath those plants (Appendix F). By contrast, although there were no significant corresponding effects for the soil phosphate fluxes, the latter were significantly correlated with the actual 2016 values for individual shrub height and lateral dimensions average (Appendix G). Soil ammonium flux was also significantly correlated with the actual 2016 values of shrub height (Appendix G). Finally, at the whole plot scale, the relative increases in birch cover were significantly and strongly correlated with plot-averaged soil ammonium flux (explaining 48% of the total variation), but not phosphate flux or soil moisture (Appendix H).

### Climate

**Figure 8:**
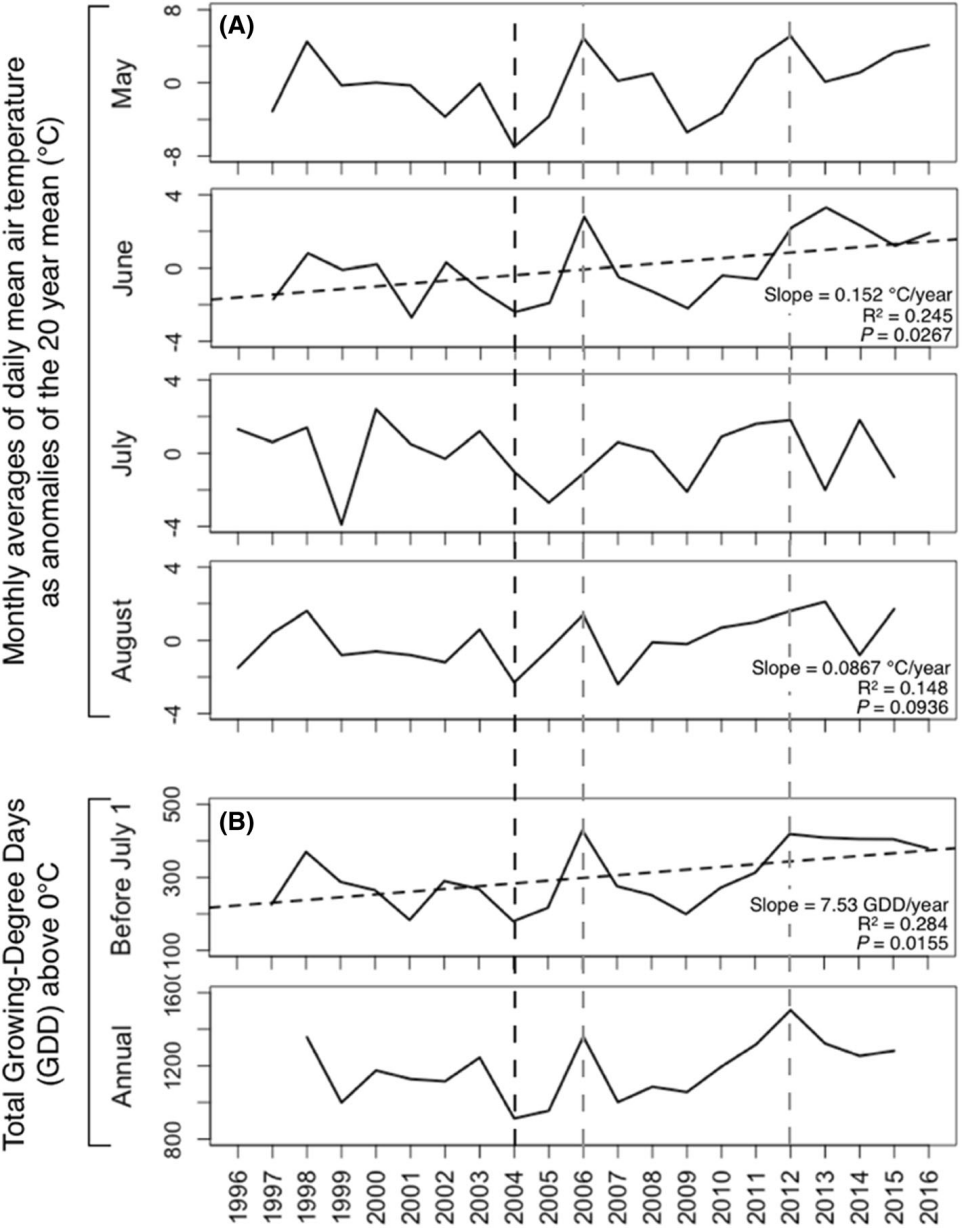
Daring Lake monthly averages of daily mean air temperature as anomalies of the 20 year means for each month from May–August 1996–2016 **A**, and total growing degree days for the periods up to July 1st each year and for the full year **B**. Linear regression lines are shown where a statistically significant trend over the 20-year time period was observed. The 2004 (black), 2006 and 2012 (both gray) dashed lines highlight relatively cool, and warm years, respectively.

The monthly averages of daily mean air temperatures over the entire Daring Lake climate record (1996–2016) increased significantly for June (by ~ 1.5°C per decade relative to the 20 year mean), and there was a corresponding statistical trend (*P* < 0.094) for August, but no trends in any other month (Figure 8; Appendix I.1). Growing degree days (GDD) up to July 1 of each year increased over the 20-year period, but there was no significant trend in total annual GDD (Figure 8). Consistent with the air temperature pattern, monthly averages of June daily mean soil temperatures at 0, 5, 10, 20 and 40 cm all exhibited similar and statistically significant rates of warming, but there were no corresponding trends in any other month (Appendix I.2). The monthly maxima of daily mean air temperatures in May and June both increased over the 20 years, but not in any other months (Appendix I.3), and furthermore, the number of days when daily maximum air temperature exceeded 20°C in June significantly increased, but not in July or August, or annually (Appendix I.4). Finally, neither monthly nor annual rainfall, nor snowmelt date, exhibited significant 20-year trends (Appendix I.5).

Because the overall conclusion above of a very limited climate warming trend is based on data from just one climate station, we evaluated the accuracy of the Daring Lake data in the context of the three nearest Environment Canada long-term climate stations (See Methods; Figure 2). Data from two gridded climate datasets (CRU TS3.10, Harris et al. 2014 and DAYMET v.3, Thornton and others 2018) were compared to the monthly instrumental records from the four stations. The temperature data for each station including Daring Lake correlated strongly and positively with the corresponding grid cell data (*r* > 0.995 and *P *< 0.001 for all relationships). Due to varied lengths of instrumental record, as well as periods of missing instrumental data, we used the CRU data to calculate long-term temperature change trends (1950–2018) for each of the four locations. We also calculated trends from 1996–2016, which includes the period of observation reported in this study as well as the decade prior. In summary, these analyses indicated that the entire region experienced a significant linear warming trend since 1950 (*P* < 0.001; Figure 2) of 0.034–0.038°C/y (a similar rate to most of the Northern hemisphere over the same period—IPCC, 2013), with winter warming being nearly three times the rate of summer. However, most importantly in the context of our study here, there were no significant trends at any of the four sites in either annual or winter temperatures for the period 1996–2016.

## Discussion

### Birch Shrub Net Growth in the Daring Lake Landscape Between 2006 and 2016

Our results clearly demonstrate substantial net growth and areal expansion of birch shrubs in the Daring Lake landscape over the decade since 2006. In answer to the first part of our* Question #1,* birch shrub cover across the landscape’s diverse habitat-types increased on average from 28% of total groundcover in 2006 to 32% in 2016, and increases relative to initial 2006 cover averaged 24%. Our repeat measurements of individual birch shrubs also indicated relative increases in height, canopy areal extent, and total primary shoot length of the same general magnitude as for overall cover. Despite substantial variability in birch cover change among plots, and in growth responses of individual shrubs, these two essentially independent datasets—measured at two different spatial scales—were remarkably consistent. First, all of the diverse metrics that we measured indicated statistically significant absolute increases over the decade, and furthermore, the relative increases in these metrics (that is, changes relative to initial 2006 values) were all of generally similar magnitude (Tables 1, 2). Second, the pattern of birch cover relative change at the whole 100 m^2^ plot scale, was closely and significantly correlated with the plot-averaged relative changes in focal shrub height, lateral dimensions average, and total primary shoot length (Figure 5). Third, all measures of relative increase at both spatial scales were greatest in the Esker Plain and Sedge habitat-types (Figure 3, Table 1).

However, in answer to the second part of* Question #1,* although there was a consistent pattern of environmental differences among habitat-types at the two spatial scales, our statistical analyses of the focal shrub data (*n* = 8) provided no evidence that habitat-type was a significant determinant of birch growth responses across the landscape. These findings align with preliminary analysis of several repeat photographs of the Daring Lake landscape (2004 and 2016) that clearly indicate some patches within a variety of habitat-types where birch shrub size and extent have substantially increased, and many other patches where there has been no observable change (Appendix J).

### What Factors Might Explain Birch Net Growth and Areal Expansion?

Below, we apply our conceptual framework (Figure 1) of the various potential explanatory factors and their principal interactions that may drive decadal patterns of deciduous shrub growth and expansion across low Arctic landscapes specifically to the pattern of results from the Daring Lake habitat-types.

#### Climate Change as a Factor Driving Net Growth

Many studies documenting recent net growth and areal expansion of deciduous shrubs across the Arctic tundra have significantly correlated those increases with warming climate (Forbes and others 2010; Blok and others 2011; Ropars and Boudreau 2012; Tape and others 2012). Furthermore, experimental warming with plastic greenhouses at Daring Lake (that raised mean air and soil temperatures across all summer months by ~ 2.2°C) doubled birch shoot biomass (Zamin and Grogan 2012), indicating that this species has the potential at our site to respond to strong prolonged growing season temperature increases. However, the 20-year Daring Lake climate station records provide only limited evidence of climate warming. Note that one of the rationales for our analysis of climate trends over the 20-year period starting a full decade *prior to* the onset of our shrub measurements is that warming can stimulate birch shrub growth even over a subsequent colder period (Kaarlejärvi and others 2015). Significant warming trends occurred only in the early growing season, and were of relatively small magnitude compared to many other, mostly more coastal, locations/shrub study sites. For example, we found a 0.15°C per year warming trend but only for the month of June. That trend averaged across the full year is equivalent to about 1/5th of the mean annual temperature increase across the entire Arctic over recent decades (Comiso and Hall 2014; IPCC 2013).

The absence of a significant influence of habitat-type in our data also suggests that climate change may not have been the primary driving mechanism for the growth increases we observed at Daring Lake. Habitat-type effects are to be expected if growth increases were being largely determined by changes in a single common factor (for example, warming) because one would expect deciduous shrubs within more fertile habitat-types across the landscape to display a combination of greater net growth, greater sensitivity of growth to annual climate, and stronger correlation of annual growth trends between shrubs (that is, more synchronous growth patterns), as compared to shrubs in less fertile habitats. In northern Alaska, shrubs in more favorable habitat-types tended to respond more strongly not just to multi-year warming trends, but also to interannual temperature variations (Tape and others 2012). In northern Quebec, dwarf birch (*Betula glandulosa*) expanded in both terrace and hilltop habitat-types since the late 1950s, but the increase was nearly twice as large on terraces (Ropars and Boudreau 2012). Shrub expansion near treeline in Siberia differed significantly among habitat-types and was largest in upland areas where permafrost-thawing processes were prevalent (Frost and Epstein 2014). However, all three of the above studies documenting differences in shrub growth among habitat-types were based in sites that have experienced substantial warming and warming-related interactive effects (for example, permafrost degradation). By reverse logic, the absence of a significant habitat-type effect at Daring Lake is consistent with our conclusion that climate warming was not the primary determinant of the pattern of birch net growth that we observed.

Our dendrochronological data also support the conclusion that the observed shrub growth patterns were not due to climate warming. If the temperature had been a primary determinant of growth, one would expect to see annual growth patterns that are shared among shrubs (at least within a habitat-type) and correlated with annual climate trends. However, in answer to our* Question #2,* we found very few common growth signals among shrubs, as indicated by the lack of correlation among ring-width chronologies between habitat-types, as well as among individual shrubs within each habitat-type (only ~ 1/3 of the samples could be confidently cross-dated). Furthermore, none of the ring-width chronologies in any habitat-type were significantly correlated with climate.

This lack of a shared climate signal in the shrub secondary growth data—a signal which has previously been clearly documented in *Betula* (Blok and others 2011; Ropars and others 2015, 2017; Trudel and Lévesque unpubl. data)—may be due to several factors. Daring Lake is far from *B. glandulosa*’s northern range limit (CAVM 2003), so it is possible that shrub growth at this relatively southerly site is less temperature-limited. In addition, our dendrochronologies were based on stem samples, and although some previous tundra studies have significantly correlated stem ring-width chronologies to climate warming trends (Ackerman and others 2017; Tape and others 2012; Forbes and others 2010), it is now known that birch root collars provide much more climate-sensitive data than stems (Ropars and others 2017).

#### Recovery Following Disturbance as a Factor Driving Net Growth

Net growth may occur as vegetation is recovering or responding to earlier disturbance events such as instances of permafrost thaw, soil active layer detachments, palsa subterranean ice accumulation/thaw cycles, fire, disease and insect outbreaks, and extreme climatic episodes. Such disturbances are naturally occurring discrete events that contribute to the long-term temporal dynamics of growth mortality equilibrium in tundra plant populations. However, there have been no significant fires, herbivorous insect outbreaks, or periods of unusually high birch mortality in Daring Lake area in recent decades (Joachim Obst (long-term TERS researcher/naturalist), *personal comm.*), and the site’s climate record contained no evidence for any prolonged period of drought or set of unusually severe winters in the past 20 years. Finally, there were no prolonged, collective periods of lower or higher growth visible in the ring-width chronologies which extend back approximately 40 years. Accordingly, we conclude that it is highly unlikely that the growth patterns observed are a recovery response to some previous disturbance event.

#### Declining Caribou Herbivore Impacts as a Factor Driving Net Growth

Caribou are an important herbivore in the central low Arctic, and Daring Lake lies in the middle of the Bathurst caribou herd’s summer range (Adamczewski and others 2019; Figure 2). Deciduous shrubs seem to constitute about 45% of caribou spring and summer diets in North America, although studies are scarce and the proportion of birch compared to willow (*Salix* spp.) is undetermined (see recent review of dietary composition studies in Appendix A of Zamin and others 2017), but probably quite low (Obst, Adamczewski, and Côté, *pers. comm.*). Nevertheless, focused studies in northern Quebec indicate that birch is a substantial proportion of the summer diet of caribou (Crête and others 1990), and that caribou herbivory can significantly reduce birch biomass and productivity in their summer ranges there (Manseau and others 1996). Furthermore, a large-scale remote sensing study that incorporated extensive on-the-ground quantification of birch (and willow) browsing damage in Nunavik concluded that intense caribou herbivory had prevented net increases in shrub cover over the past 40 years, even though there had been rapid climate warming in that region over that period (Plante and others 2014). At Daring Lake, Zamin and Grogan (2013) found that caribou exclusion from about 400 m^2^ plots for 5 years (between 2004 and 2009) resulted in a doubling of birch leaf biomass, and similar results have been reported from experimental reindeer exclosures in N. Sweden (Olofsson and others 2009). Together, these results strongly suggest that herbivory and trampling by caribou—especially when they are in high numbers—can be an important constraint on low Arctic tundra birch growth. However, like most other major N. American caribou herds (Fauchald and others 2017), the Bathurst herd has declined severely in recent decades, from an estimated 470,000 in 1986 to 32,000 in 2009 and most recently to 8200 in 2018 (Adamczewski and others 2019). Effects of this particular caribou herd decline are visibly apparent at Daring Lake—for instance, our repeat photography clearly illustrates birch shrub expansion within *some* landscape patches and also some recent birch encroachment across caribou trail pathways (Appendix J).

We conclude that the release from these herbivory and trampling pressures due to the severe ongoing reduction in the Bathurst caribou herd is the most likely explanation for the net growth and expansion of birch we observed over the 2006–2016 decade. First, caribou are probably the principal birch herbivore in this low arctic landscape—arctic hares definitely significantly browse individual birch especially close to the eskers (pers. obs.), but have strong habitat-type preferences. Second, and by contrast, caribou are constantly moving through the entire landscape, and so their herbivory and trampling are likely to be spatially uniform over decadal timescales, thereby affecting birch shrubs in multiple different habitat-types, as has been previously observed in Quebec (Manseau and others 1996).

Why did we not detect an increase in ring-widths following the presumed release from caribou herbivory? Arctic birch stem and root collar ring-width growth can respond to climate warming (for example, Forbes and others 2010; Ropars and others 2015), and it is known that herbivory pressure can significantly influence stem secondary growth rates of other *Betula* species (Speed and others 2011). One possible explanation is that birch shrubs have a relatively strong ability to quickly divert resources to the conversion of short lateral sideshoots to long shoots under favorable conditions such as when soil fertility is enhanced (Bret-Harte and others 2001), which may result in an increase in apparent stature without substantial thickening of the main axis. However, since warming stimulates both lateral sideshoot growth (Bret-Harte and others 2001) and stem and root collar secondary growth (Forbes and others 2010 and Ropars and others 2015, respectively), this explanation requires that there be a substantial allocation difference in birch growth responses to release from herbivory compared to warming. Compensatory growth in the form of rapid extension of lateral short shoots following simulated caribou herbivory has been observed in birch (Champagne and others 2012), supporting the possibility that release from herbivory could have stimulated new lateral shoot production at our site with no major change in main shrub axis thickening rates.

### Factors Affecting Individual Shrub Growth Across the Landscape

Although a release from caribou herbivory and trampling appears to be the principal factor driving the overall net increase in birch shrub stature and cover across the Daring Lake landscape, our soil environmental data indicate that other factors significantly affected the growth rates of individual shrubs. In answer to* Question #3,* soil ammonium flux, in particular, was positively correlated with the relative increases in all three stature metrics of individual shrubs (Appendix F), and to the overall relative increases in birch cover in the 100 m^2^ monitoring plots (Appendix H). The consistency of these correlations for all measured variables from two essentially independent datasets at two different spatial scales, at least suggests that increases in soil nitrogen availability may have been a contributory causal factor in determining the birch relative growth responses. In other words, either release from herbivory, and/or June warming, may have enhanced soil nitrogen availability beneath some individual shrubs, resulting in the observed variation in individual birch relative growth responses. By contrast, although there were no significant corresponding effects for the soil phosphate fluxes, the latter were significantly correlated with the 2016 values for birch height and lateral dimensions average (Appendix G). Likewise, the soil ammonium fluxes were significantly correlated with the 2016 birch height values (Appendix G).

Given that experimental factorial fertilizer additions have previously demonstrated that birch shrub growth in at least mesic tussock tundra at our site is strongly co-limited by both nitrogen and phosphorus (Zamin and Grogan 2012; Zamin and others 2014), it is not surprising that in situ soil fluxes of both nutrients should be correlated with birch growth dynamics and stature variables. Perhaps more surprising is that in situ fine-scale variability in these fluxes across the landscape was such an important determinant of those growth responses. Individual shrubs that were even short distances apart (that is, within the same plot) often had very different nutrient flux rates in their underlying soil, and there were no overarching differences in mean soil fertility among habitat-types. Even those habitat-types with the lowest average ammonium fluxes (Esker Plain and Tussock) had occasional shrub locations with relatively high fluxes, and likewise for phosphate (Figure 7). Together, these results imply that the growth rate of an individual shrub following herbivore release is determined more by its precise location on the landscape, and the amount of soil resources available in its immediate surroundings, than the habitat-type that the shrub resides in. This conclusion suggests, therefore, that in contrast to the important influence of habitat-type in predicting shrub growth responses to climate warming (for example, Tape and others 2012; Ropars and others 2015; Blok and others 2015), it has little influence in predicting shrub growth responses to herbivory release. Instead, much finer-scale spatial variability in soil ammonium fluxes seems to determine the relative growth responses of individual shrubs to release from herbivory. Furthermore, likewise, fine-scale spatial variability in both ammonium and phosphate fluxes correlates with the absolute values of birch height across the landscape (and phosphate fluxes also seem to be a strong determinant of shrub lateral dimensions).

### Implications

This study demonstrates substantial net birch growth and cover expansion across the Daring Lake landscape over the 2006–2016 decade, when there was only limited evidence of climate warming but a severe decline in the caribou herd. Although not absolutely conclusive, our study’s results suggest the potential for caribou herbivore population declines to significantly contribute to increasing deciduous shrub abundance across the Arctic. If correct, the shifts in vegetation should be strongest in tundra areas with the most drastic declines in caribou populations, and so a quantitative analysis of herd decline rates versus average range satellite-based greening rates across the Arctic would be a fascinating research extension of our study. In terms of future impacts, many caribou populations are declining globally, driven by a number of threats which are likely to worsen as climate change continues (Fauchald and others 2017). Furthermore, an experimental study has already demonstrated that birch growth responses to warming can be enhanced by excluding reindeer (Olofsson and others 2009). If deciduous shrub expansion in response to release from caribou herbivory and trampling is widespread, future Arctic vegetation change (and consequently, decreased land surface albedo and greater CO_2_ release from Arctic soils) will be substantially greater than would be expected from warming alone, particularly in regions where warming is not yet substantial. Finally, in terms of modeling future tundra vegetation change, our results suggest that the significant influence of habitat-type on deciduous shrub expansion in response to climate warming may not apply in predicting shrub change as a result of declining caribou herds.

## Electronic supplementary material

Below is the link to the electronic supplementary material.

Supplementary material 1 (PDF 8981 kb)
