# Supplementary material for: Recent Growth and Expansion of Birch Shrubs Across a Low Arctic Landscape in Continental Canada: Are These Responses More a Consequence of the Severely Declining Caribou Herd than of Climate Warming?
Source: Ecosystems. 2020 Jan 9;23(7):1362–79. doi: 10.1007/s10021-019-00474-7 (PMC7666286; doi:10.1007/s10021-019-00474-7)
Supplement: Supplementary file 1 — Supplementary material 1 (PDF 8981 kb) [file 10021_2019_474_MOESM1_ESM.pdf]

Appendix A1. The principal habitat-types where birch typically occurs in a low Arctic landscape such as around Daring Lake, and their defining characteristic features. Parentheses indicate data from measurements in the Daring Lake habitat-type plots (see Methods for details).

Habitat-type	Defining topographical location	Soil description (Mean and S.E. of late summer active layer depth)	Soil moisture regime across the growing season	Wind exposure	Winter snow accumulation (Minimum mid-winter snow depth estimate)	Dominant vegetation
**Esker Plain**	Esker and hilltop ridges	Thin (1-5 cm) organic layer above coarse-grained mineral or gravel deposits (36 cm +/- 3.8)	Low	High	Low (10 cm)	Lichen heath: sparse, well-developed lichen layer with several evergreen species and occasional low-lying birch shrubs
**Sedge**	Low-lying valley bottoms and landscape depressions	Thick organic active layer (50 cm +/- 4.8)	High – generally inundated	Low	Moderate (35 cm)	Wet sedge: strongly dominated by sedges with occasional birch shrubs
**Snowpack**	At the base sides of eskers and hilltop ridges; topographic channels and grooves	Variable (1-5 cm) organic layer above coarse-grained mineral deposits (53 cm +/- 7.0)	Moderate	Moderate	High (90 cm)	Tall Birch: dense birch shrub cover, with a variety of evergreens and mosses in the understory
**Tussock**	Low-lying plateaus at	Top surface organic layer 5-	Moderate-to-High,	Moderate-to-Low	Moderate (35 cm)	Birch Tussock: evergreen and deciduous shrubs, forbs,

	elevations above sedge	12 cm deep above mineral deposits (66 cm +/- 8.0)	but generally not inundated			mosses and tussock-forming sedges
**Watercourse**	Adjacent to rivers, streams, or subterranean water channels	Moderate (1-15 cm) organic layer above coarse-grained mineral deposits (58 cm +/- 13)	Moderate	Moderate	Moderate-to-High (49 cm)	Tall birch: dense birch shrub cover with extensive underlying moss cover

Appendix A2. Photos (and GPS locations) of the duplicate long-term monitoring plots for each major birch habitat-type across the landscape near Daring Lake, N.W.T., Canada.

Esker plain
(64.8718 -
111.5631;
64.8725, -
111.5825)

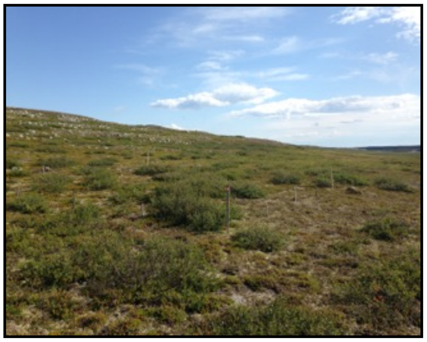
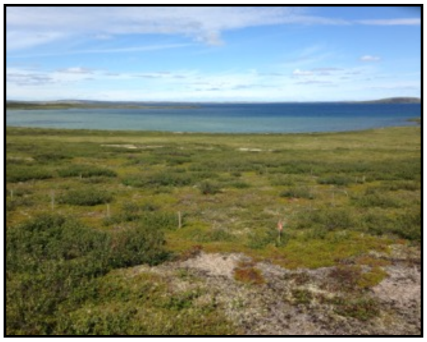

Sedge
(64.8669, -
111.5534;
64.8671, -
111.5559)

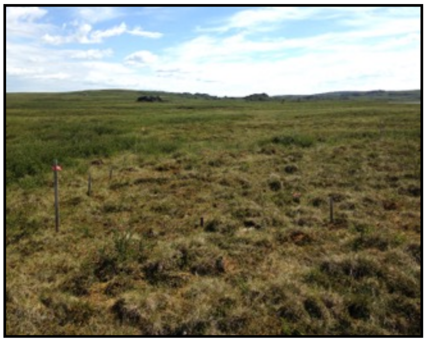
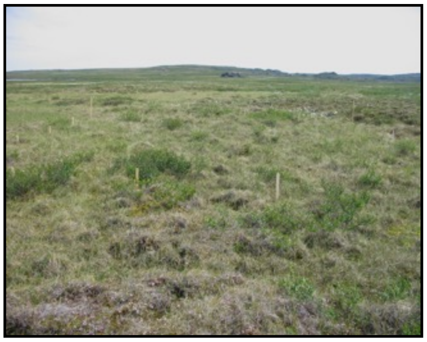

Snowpack
(64.8724 -
111.5607;
64.8745, -
111.5538)

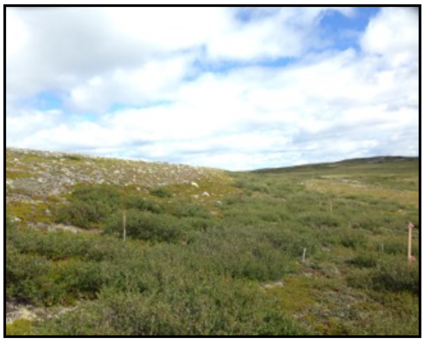
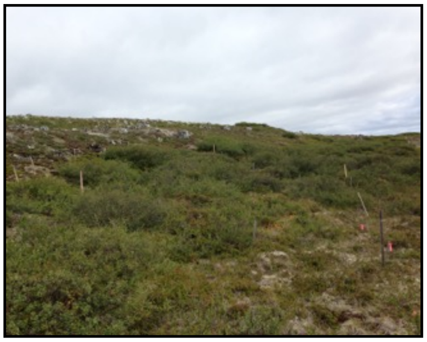

Tussock
(64.8699, -
111.5569;
64.8673, -
111.5564)

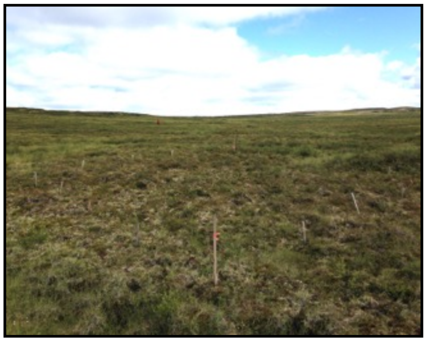
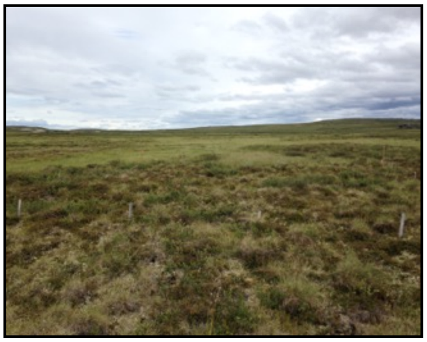

Watercourse
(64.8825, -
111.6022;
64.8738 -
111.5606)

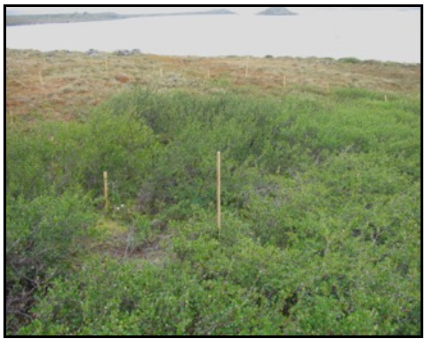
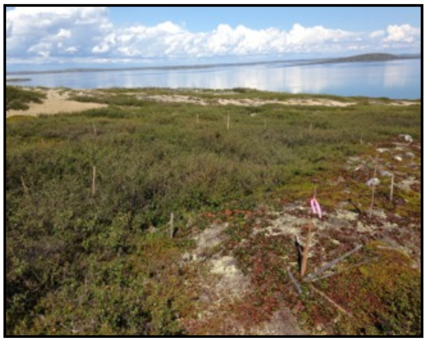

Appendix B. Shrub Cover-map Validation Procedure

The birch ground-cover maps for each of the ten 100 m^2 long-term monitoring plots used in this study were generated by two different researchers, ten years apart, and so it is critical to verify that the drawing abilities of the two researchers did not generate a bias in the cover map data (e.g. if one researcher tended to draw shrubs larger than the other, this would result in an inherent bias toward either more or less shrub-cover increase than actually occurred). To evaluate this potential bias, the four focal shrubs at each observational plot were identified on the hand-drawn cover maps, based on indications drawn during the generation of these maps. Out of the 40 focal shrubs, 16 were distinct individuals whose areal extent was clearly distinguishable from other shrubs in 2016 (the rest were located within a continuous or semi-continuous area of cover, where the land-cover of a single shrub was not easily distinguished). For each of these distinct shrubs, their lateral dimensions in the x and y directions relative to the observational plot were inferred based on the 2016 cover maps using a ruler (as the maps were drawn at a constant scale). This same process was then performed for these same shrubs based on the 2006 maps.

Using these inferred measurements, an average % lateral dimension change based on the cover maps was generated for each of these shrubs. This was compared to the actual average % lateral dimension change, based on direct measurements of these shrubs (see "Methods"), and the difference between these two values was calculated for each shrub. Using a one-sample t-test, it was determined that the mean of these differences in average % lateral dimension change between the cover maps and physical measurements were not significantly different from zero ($t = 0.669$, df = 15, $P = 0.514$). This result indicates that the 2006-2016 decadal changes in the dimensions of the shrubs derived from the ground-cover maps were not significantly different from than the changes in the actual *in situ* measured dimensions of those shrubs. Therefore, we

can assume that either both researchers were able to accurately draw shrubs to true size, or else that the bias towards drawing larger or smaller shrubs was shared to a similar extent between researchers (and thus the net difference between years would be unaffected).

Appendix C. Photos of a birch shrub stem section being cut with a microtome (a), cut sections being stained with Toluidine Blue to improve ring visibility (b), stem cross-sections (seen through a microscope) with visible growth-rings (c—e), and a stem cross-section with visibly uneven growth-rings, which were present in some shrubs (f).

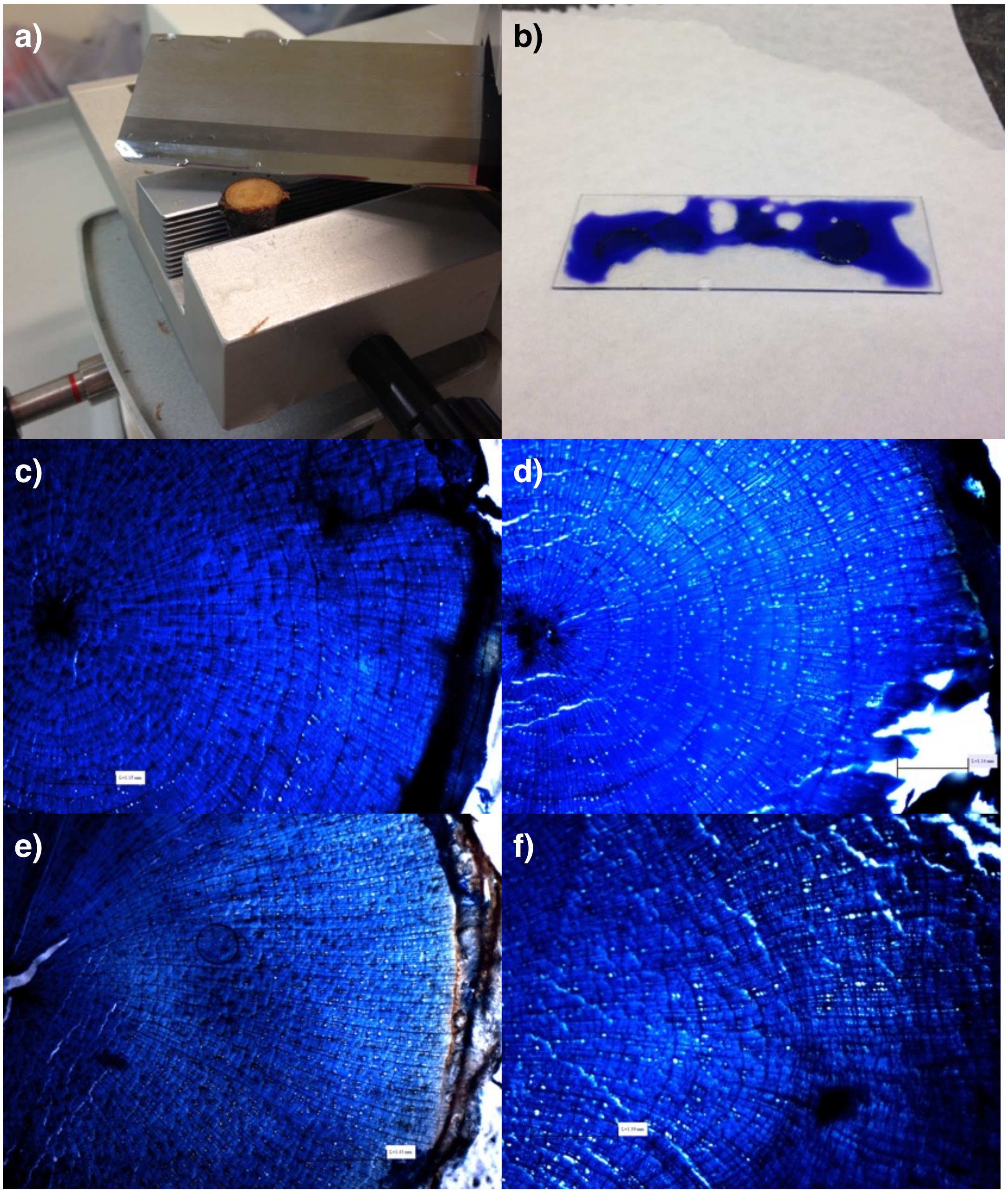

Appendix D. Ion Exchange Membrane (IEM) Preparation, Charging, Insertion, Removal and Elution Procedure

Materials for preparation and insertion:

- Gloves
- Cation and Anion IEM sheets, in sealed packaging from manufacturer
- Marker
- Knife
- Ruler(s)
- Clothespins
- Distilled water
- Plastic containers, large enough to allow full submersion of IEM strips, and made of either polypropylene or high-density polyethylete
- Large ziplock bags
- 0.5M HCl
- 0.5M $NaHCO_3$
- Tagging gun + tags (two different colours)
- Flagging tape
- Cooler + Ice packs (for transport)
- Large, wide knife (bread or steak knife) (for IEM insertion)

Procedure for IEM preparation and insertion:

1.) Cut the IEM sheets

Lie the IEM sheets out on a clean lab countertop and pin the layers of each sheet together with clothespins to keep them from separating during the cutting procedure. Using a ruler and marker, mark out 5x5 cm sections onto the plastic packaging. Using a clean, sharp knife or scissors, cut the IEM sheets along the marker lines until all sheets are sectioned into 5x5 squares. Ensure that the knife/scissors are well cleaned between different membrane types. Immediately after cutting, place membranes into a large plastic contained with 2L distilled water (do not mix membrane types) to keep sheets hydrated. Thoroughly rinse each sheet individuals with distilled water, and store sheets in a clean, sealed plastic bag with a small amount of water, kept in a refrigerator until charging.

2.) Charging cation IEMs

Charging of IEM strips should occur as close to the insertion date as possible. Remove cation IEM strips from storage. Pour an adequate volume of 0.5M HCl into a large plastic

container to cover the IEM strips (multiple containers may need to be used depending on number of samples). Place the lid on the container and gently shake container (leaving the container on the countertop) for 10 minutes. Ensure that all IEMs are submerged, and leave container to soak for one hour. Prepare two other large plastic containers, one with distilled water and one with fresh 0.5M HCl (using the same volume as the first HCl bath). One-by-one, remove IEM strips from the HCl bath, rinse each strip in distilled water, and then place into the fresh HCl. Repeat the same shaking and soaking procedure as the first HCl bath. Repeat procedure until IEM strips have undergone three HCl baths. Rinse all IEMs in distilled water one last time, and store all IEMs in a fresh, clean ziplock bag with a small volume of distilled water to keep membranes hydrated. Store in the fridge or in a chilled cooler until insertion. Prior to insertion, leave a few charged strips in the fridge to use as blanks.

3.) Charging anion IEMs

Follow a similar procedure to step 2.), but using the anion IEM sheets, and 0.5M $NaHCO_3$ instead of HCl.

4.) Inserting IEMs in the field.

Just prior to insertion, tag each IEM strip with the tagging gun. This can be done by first rinsing the tagging gun needle and tags in distilled water, and loading the gun with tags. Place the IEM strip against a clean surface with a hole in it for the tagging gun needle to go through (e.g. a pipette holder) and insert the tagging gun needle through the corner of the IEM strip. Pull the trigger to insert a plastic tag through the IEM. Place IEMs into ziplock bags with a small amount of distilled water to keep them hydrated, storing them in the fridge until insertion.

At each selected location, remove an IEM strip from the ziplock bag (being sure to wear gloves). Select the area where the IEM will be inserted. Using the large, flat knife, cut a slot into

the soil roughly two inches wide, and wiggle the knife back and forth to widen this slot such that an IEM can be inserted. Insert the IEM to a minimum depth of 2cm, leaving the plastic tag sticking out above the soil surface. Take a piece of flagging tape (being sure to colour-code cation and anion IEMs using different colours of flagging tape) and tie it to the plastic tag to label the IEM's location. Press firmly on the soil surface to ensure full contact between the soil and the IEM. Note that in some locations (e.g. on the top of an esker), the soil may not be deep enough to insert IEMs vertically; if this is the case, cut the slot at an angle such that the entire IEM can be buried beneath the soil surface.

Ion Exchange Membrane (IEM) Removal and Elution Procedure

Materials for removal and elution:

- Gloves
- Bench coat/paper towel
- Ziplock bags
- Permanent marker
- Distilled water
- Squeeze bottle (for rinsing in the field)
- Scissors/knife
- Cooler + ice packs
- Blank IEM strips
- Shaker table
- 2M NaCl + 0.1M HCl
- Petri dishes
- Elastic bands
- Erlenmeyer flasks with Buchner funnels
- Fine-grained fiber filter paper (for Buchner funnels)
- Vacuum filter
- Pipettes
- Small plastic vials (e.g. AA3 vials)
- Waste solution container

Procedure for removal and elution:

1.) IEM Removal

Once the IEMs have been *in situ* for the appropriate amount of time, retrieve all IEMs (this should ideally be done on the same day, and in the same order that they were inserted). Using the plastic tag, pull the IEM out of the soil, and rinse it in distilled water using the squeeze bottle to remove all soil particles. Place the IEM into a prelabeled ziplock bag, and store these

bags in the freezer until elution. Also bring the blank IEMs into the field, rinse them with distilled water, and store them with the other IEMs (this accounts for contamination from the transport and rinsing procedures).

2.) IEM Elution

Set up a clean area in the lab with paper towel or bench coat (also be sure to wear all PPE). One-by-one, remove either the cation or anion IEMs from their ziplock bags (do one type at a time, cleaning all labware in between) and place them into a clean petri dish. If using two cation or anion replicates per site, you may place both IEMs into the same petri dish (this may be beneficial if expected nutrient concentrations are very low). Place 12mL of 2M NaCl + 0.1M HCl solution into the petri dish (it should be just enough to cover two IEM sheets; adjust accordingly if using more or fewer sheets). Place rubber bands around the petri dish to keep it shut, and place petri dish onto a shaker table. Once all IEMs are soaking, start the shaker table and set it to a gentle speed. Leave petri dishes on the shaker table for a minimum of two hours.

While samples are eluting, set up buchner filter flasks for filtration. Once shaking is completed, remove all petri dishes from the shaker table. One-by-one, remove eluted IEMs and store them in ziplock bags in the freezer (in case a second elution is needed) and pour the solution from the petri dish into the buchner funnel, using the vacuum filter to filter it through the filter paper and into the ehrlenmeyer flask. Ensure that there are no particles or visible debris left in the sample (a second filtration may be necessary if this is the case). Pipette sample into small plastic vials, in appropriate volumes for further analysis, and freeze until future use.

Appendix E. Estimated Average Minimum mid-Winter Snow Depth

In plots where snow observations had not been made previously, we estimated the minimum snow depth present at a particular point on the landscape during the time of peak mid-winter snow. Blowing snow tends to accumulate against raised topographic features (hills, eskers, etc.) in all directions, completely filling in depressions on the landscape. Therefore, the relative "depression" of a shrub's location against a landscape feature represents a rough estimate of how much snow might be expected to accumulate at that location.

To estimate the "depression" at each shrub on the landscape, the nearest dominant topographic feature to each shrub was identified (i.e. a ridge or hill). One observer stood at the location of the shrub, and a second observer lay down on the ground on the opposite side of the shrub from the dominant topographic feature at the point where a snowbank would be expected to start accumulating against the identified landscape feature (usually, several meters away from the top of this feature). The observer standing at the shrub held a meter stick pointing straight up from the ground, and the observer lying down indicated to the first observer the point along the meter stick at which the top of the landscape feature visually intersected the meter stick (Fig. E a,b). The distance from the ground to the intersect is an estimation of the minimum amount of snow that would be likely to accumulate at this particular spot in mid-winter.

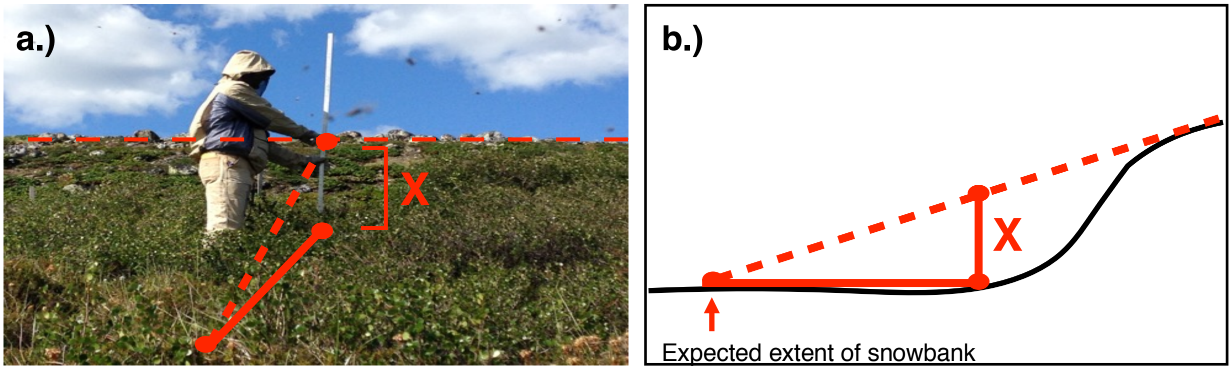

Figure E. An illustration of the snow depth estimation procedure, from the observer's view (a) and from a side view (b). The distance labelled X indicates the estimated average minimum snow depth. Diagrams are not to scale.

Appendix F. Relative changes from 2006-2016 in birch shrub height (a, b), lateral dimensions average (c, d), and total primary shoot length (e, f), in relation to soil ammonium and phosphate fluxes (µg per cm^2 per day) for each focal shrub across all habitat-types at Daring Lake (n=40). Linear regression lines are shown where significant relationships were observed.

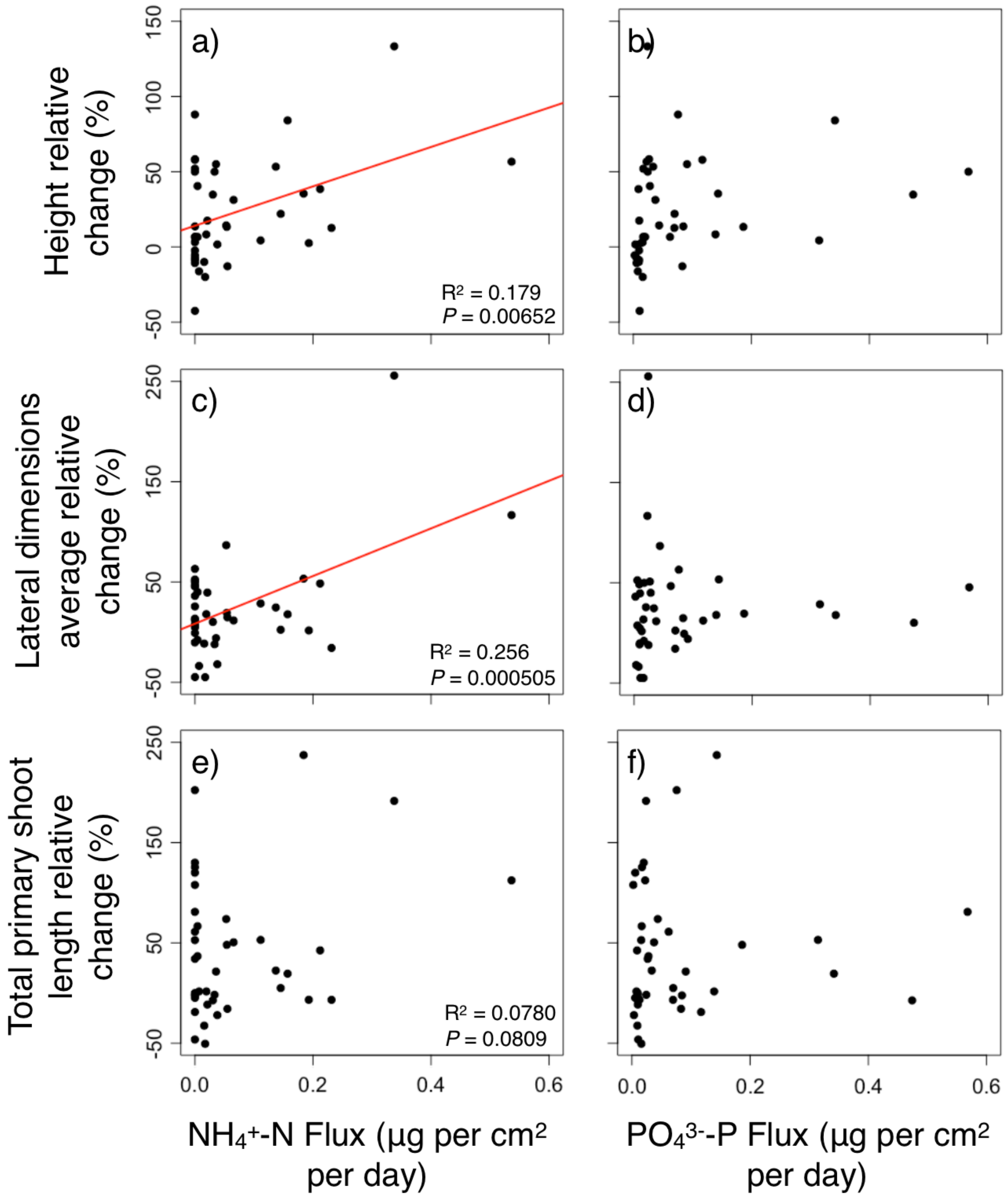

Appendix G. Soil ammonium (a—c) and phosphate (d—f) fluxes (µg per cm^2 per day) in relation to height, lateral dimensions average, and total primary shoot length for each focal shrub across all habitat-types at Daring Lake in 2016 (n=40). Linear regression lines are shown where a significant relationship was observed.

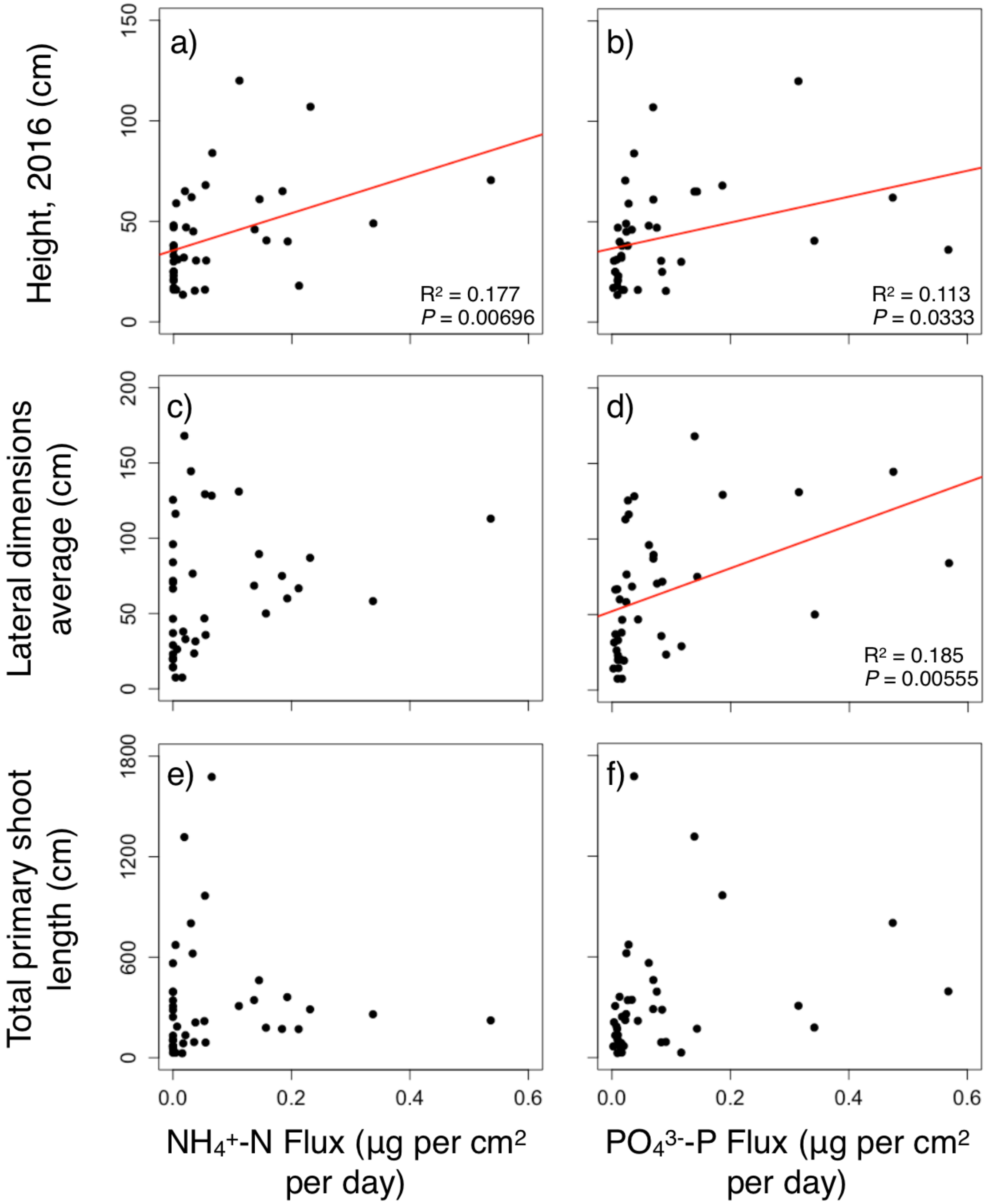

Appendix H. Relative change in birch shrub ground-cover (defined as the % change in cover between 2006-2016, divided by % cover in 2006) in each long-term monitoring plot (100 m^2; n=10) in relation to plot-averaged mean July-August soil % moisture (a), ammonium flux (b), and phosphate flux (c). Linear regression lines are shown where significant relationships were present.

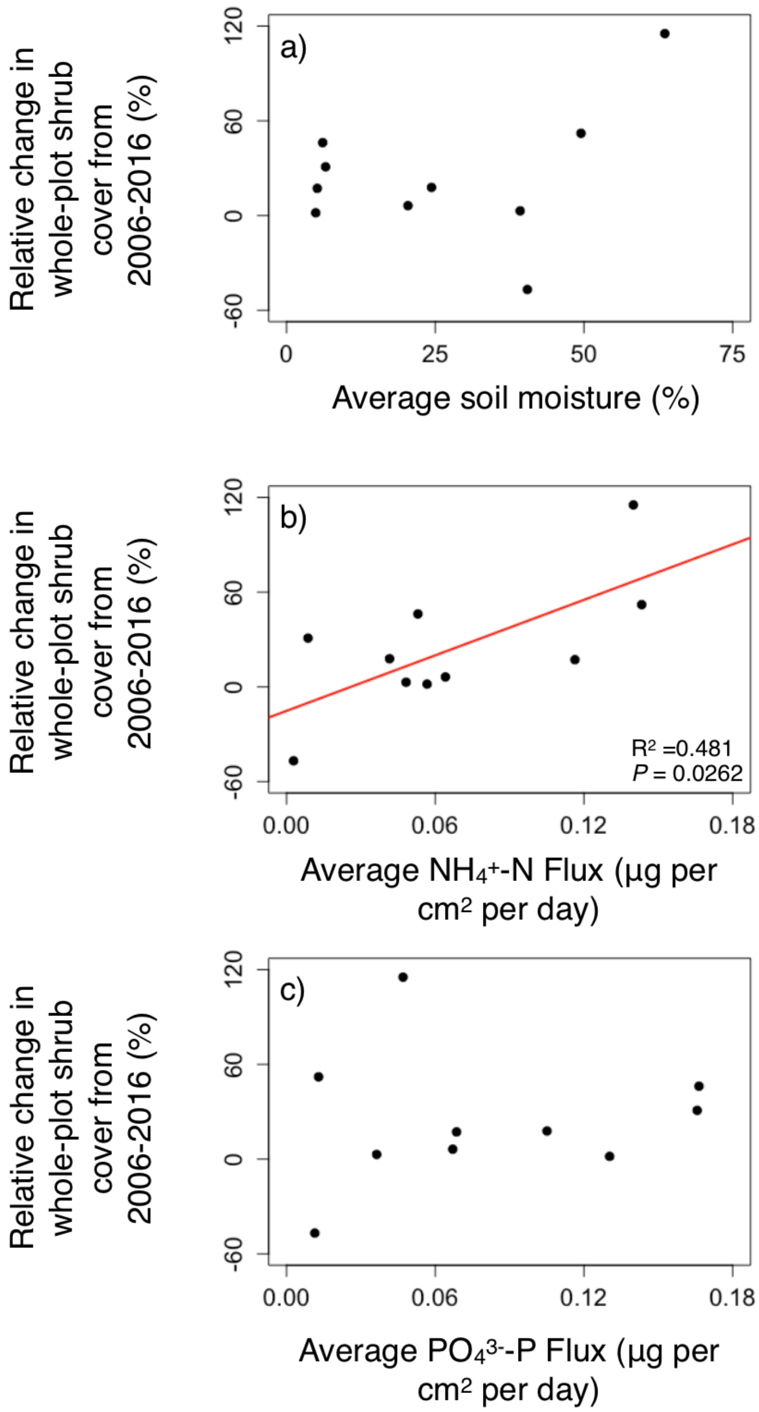

Appendix I.1. Monthly averages of the daily mean air temperatures (°C) at Daring Lake, for all months, 1996-2016. Some data are missing because of climate station malfunctions. Linear regression lines are shown where a statistically significant change over time was observed. Note that the regression values here differ from those in Fig. 8 in the main manuscript because they are based on actual means rather than anomalies.

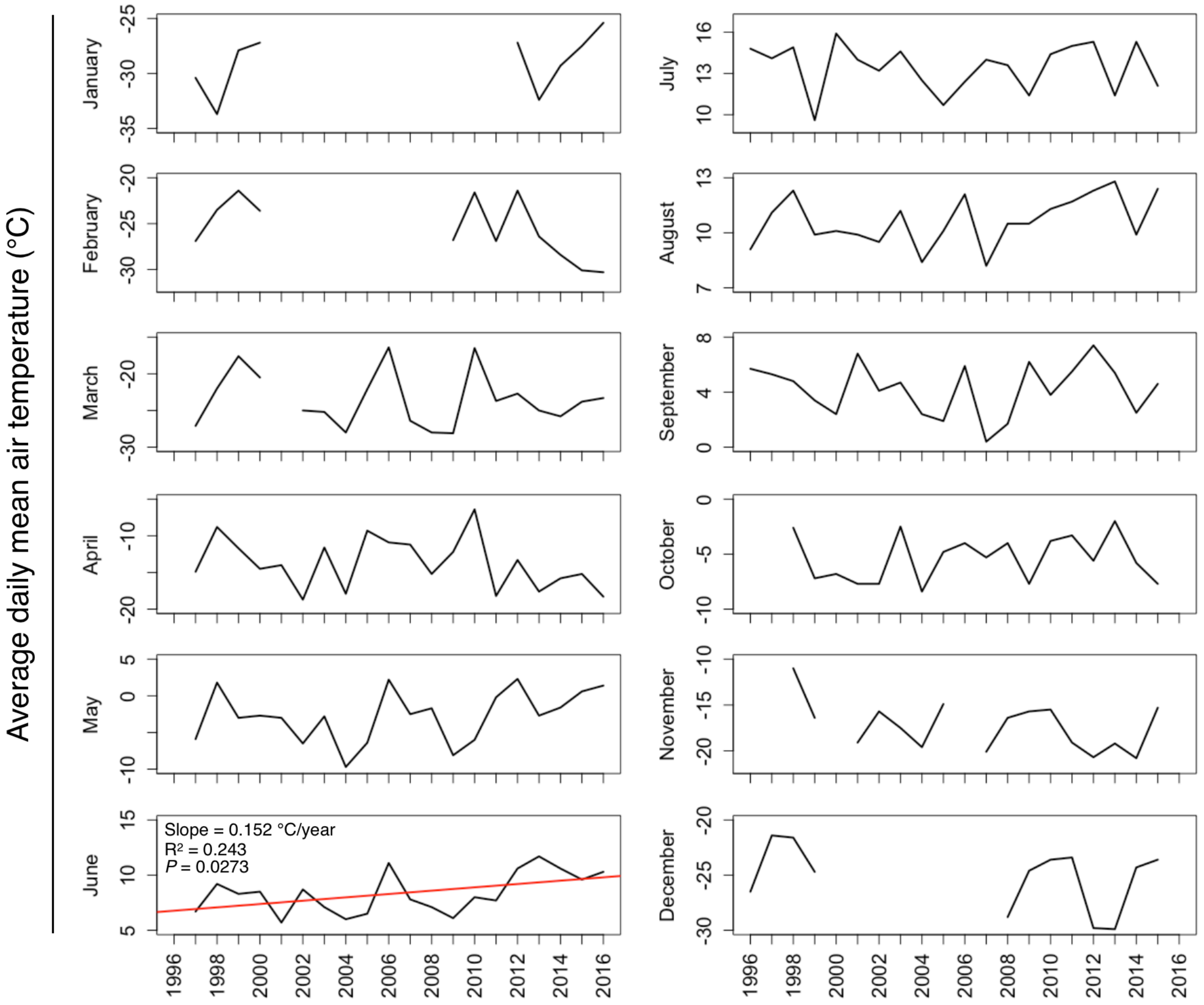

Appendix I.2 Monthly averages of the mean daily soil temperatures (°C) at a variety of depths, at Daring Lake, May-August, 1996-2016. Linear regression lines are shown where a statistically significant change over time was observed. Data for August 2012 are missing because of a logger malfunction.

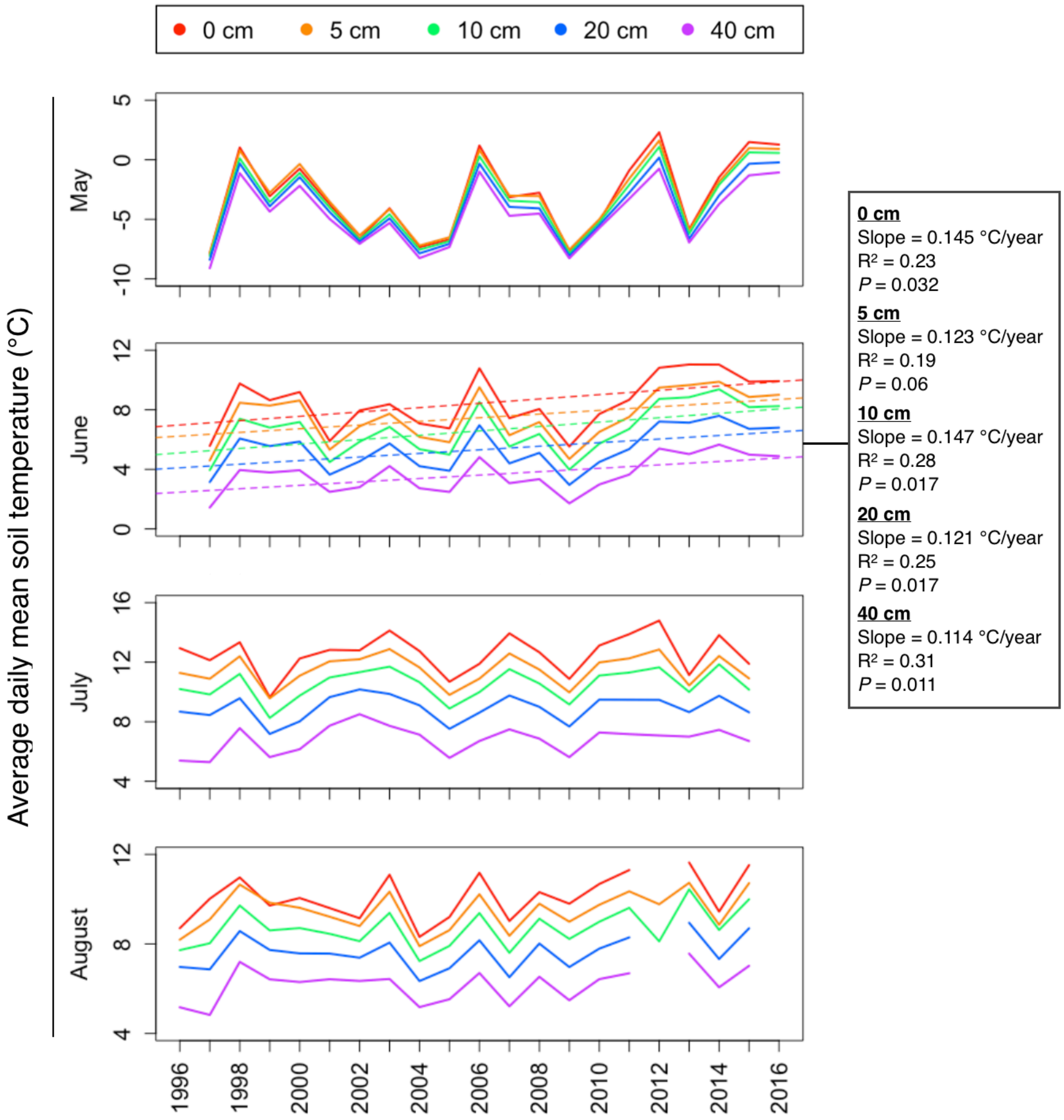

Appendix I.3. Monthly maxima of the daily mean air temperatures (°C) at Daring Lake from 1996-2016 for April-August. Linear regression lines are shown where a statistically significant change over time was observed.

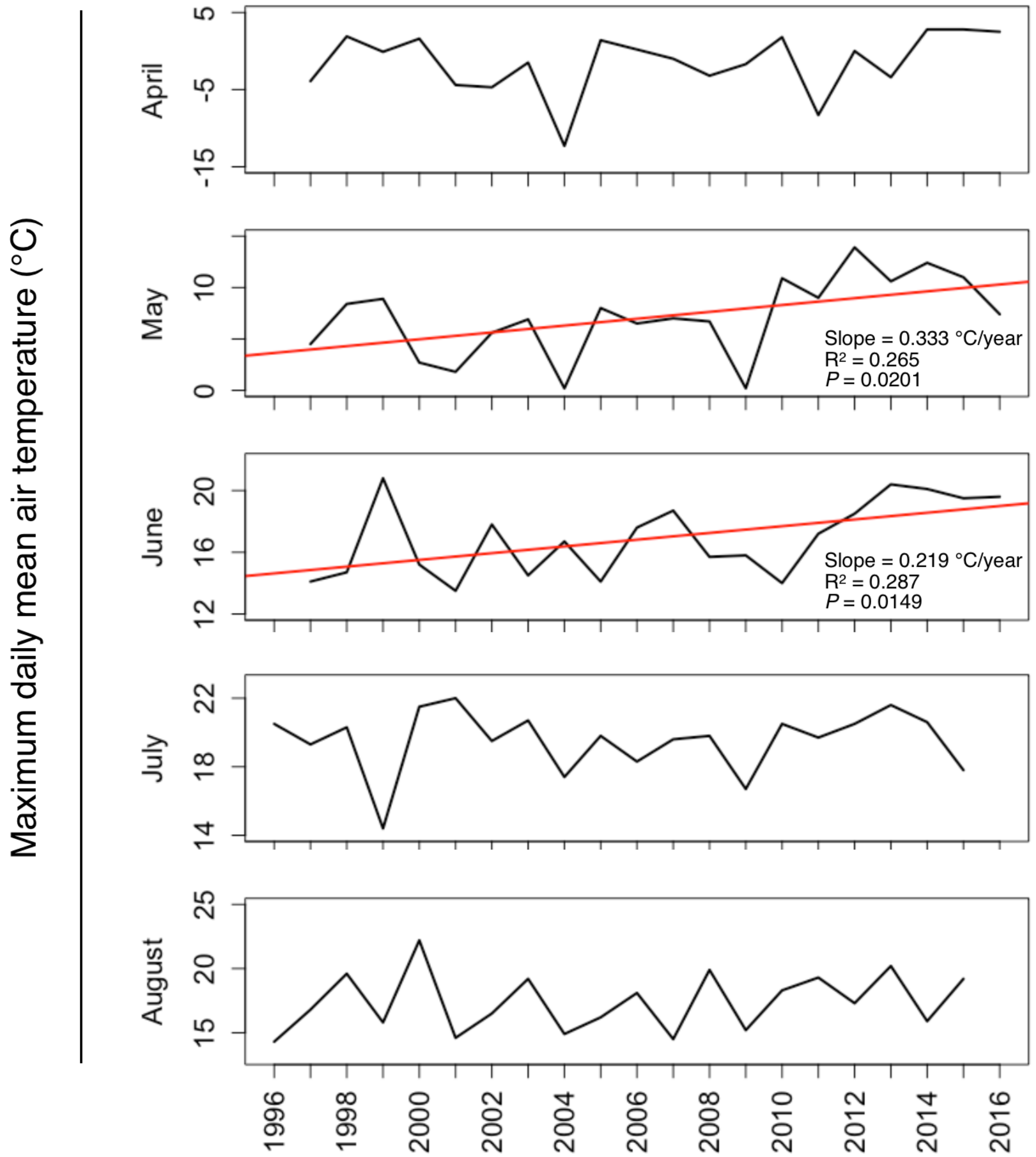

Appendix I.4. Total number of days per month with a maximum air temperature above 20°C for June-August (a-c) and annually (d) from 1996-2016 at Daring Lake. Linear regression lines are shown where a statistically significant change over time was observed. Some data for 2012 and 2013 are missing due to logger malfunction.

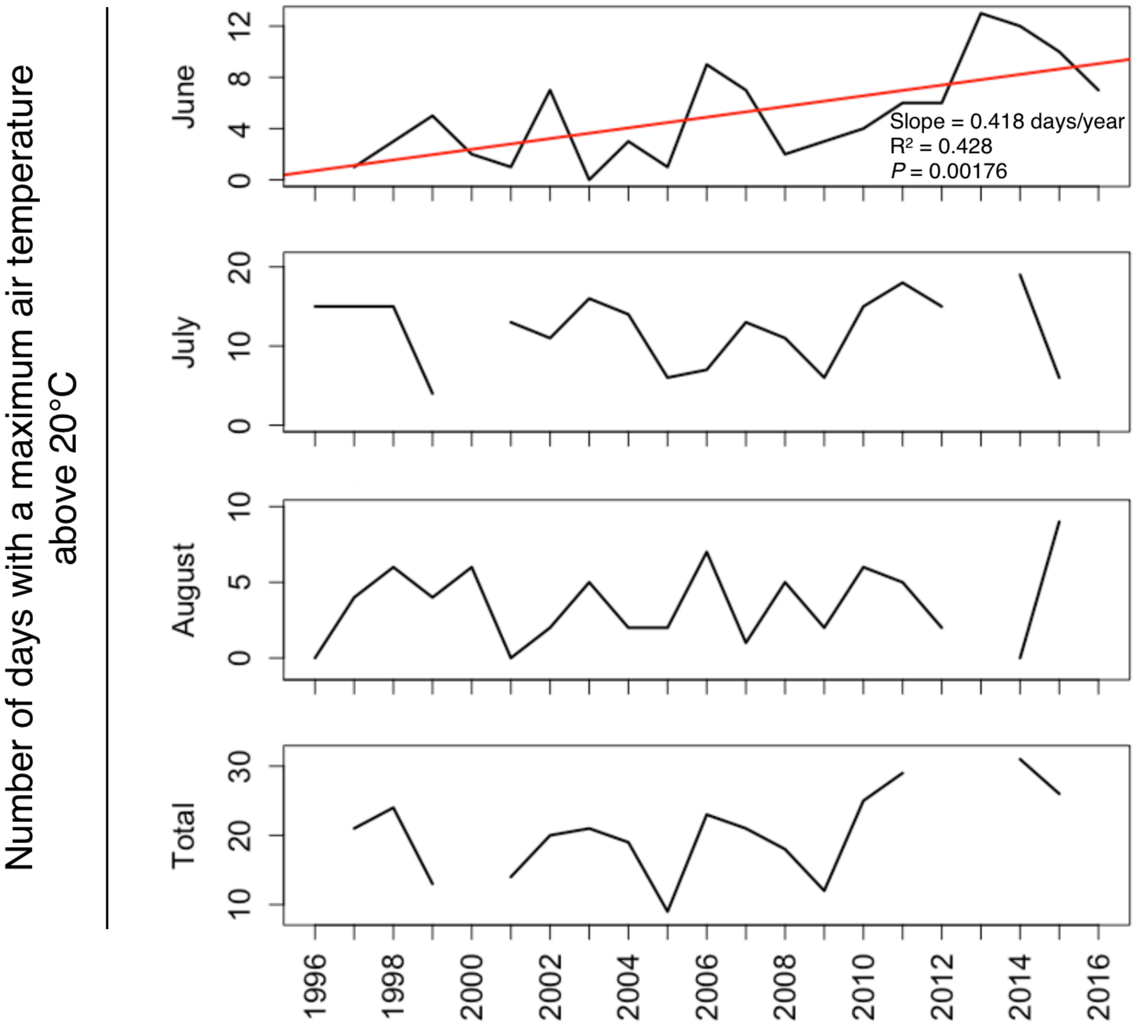

Appendix I.5. Snowmelt date (a) and total rainfall (mm), both monthly for May-August (b-e), and annually (f), at Daring Lake, 1996-2016. No significant linear trends over time were observed. Data for August 2012 are missing because of a logger malfunction.

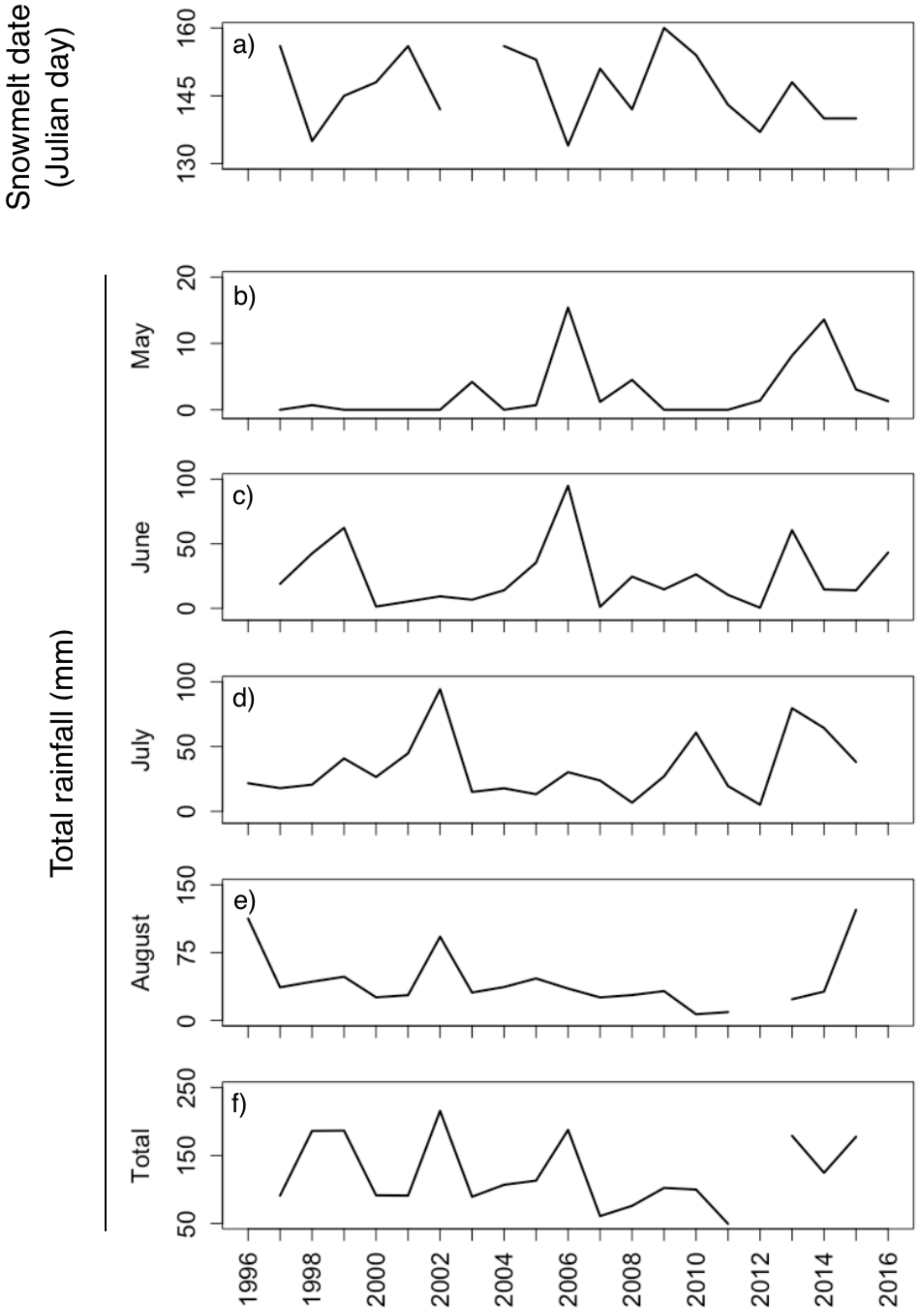

Appendix J. Preliminary repeat photo analysis of birch shrub cover increases in various habitat-types of the Daring Lake landscape

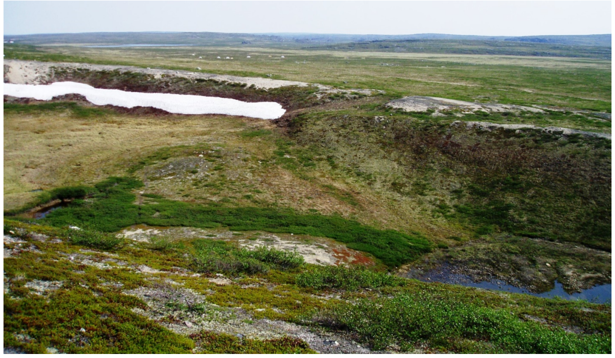

July 7, 2004

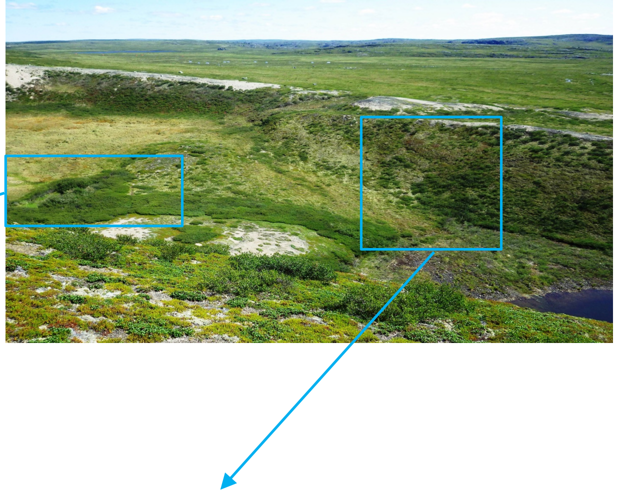

See next page

July 8, 2016

Snowpack birch habitat

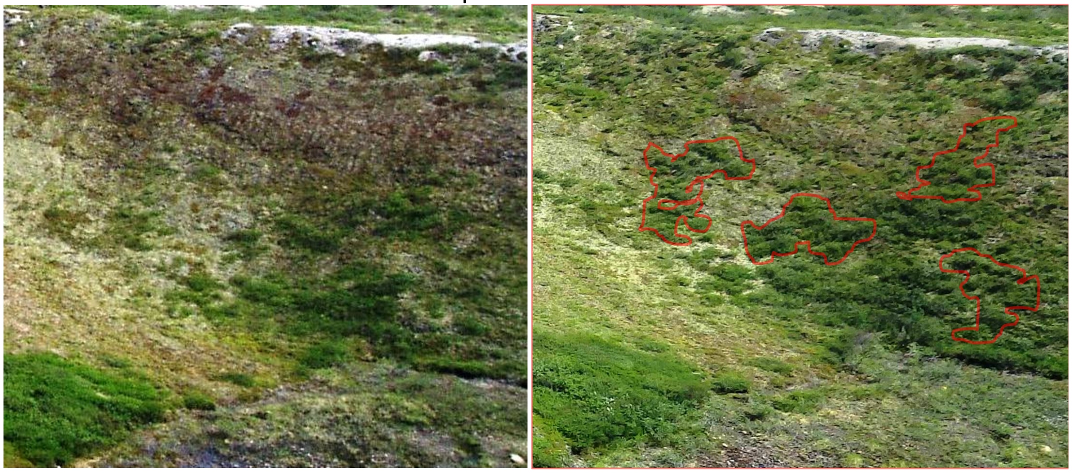

July 7, 2004 July 8, 2016

Watercourse habitat of mainly birch with some willow that includes a caribou trail:

July 7, 2004

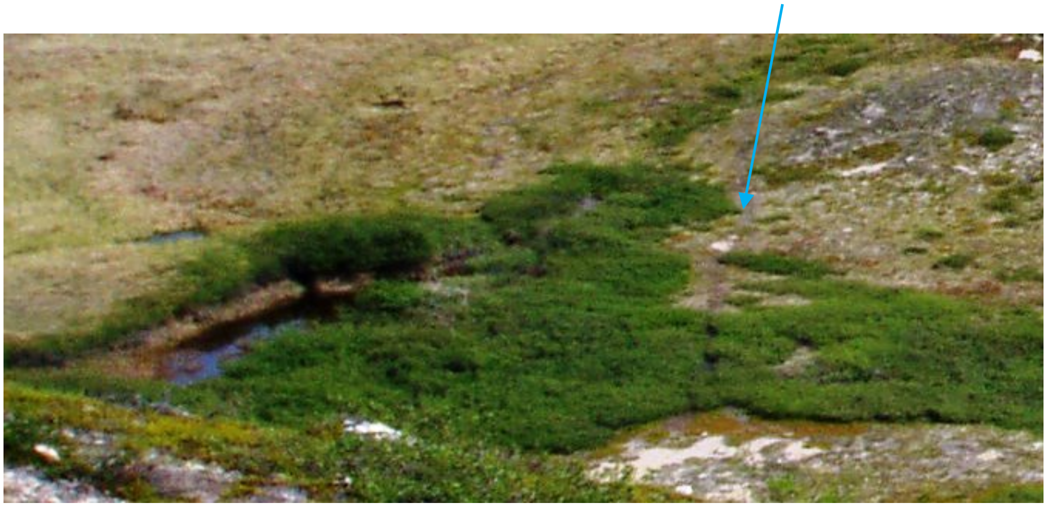

July 8, 2016

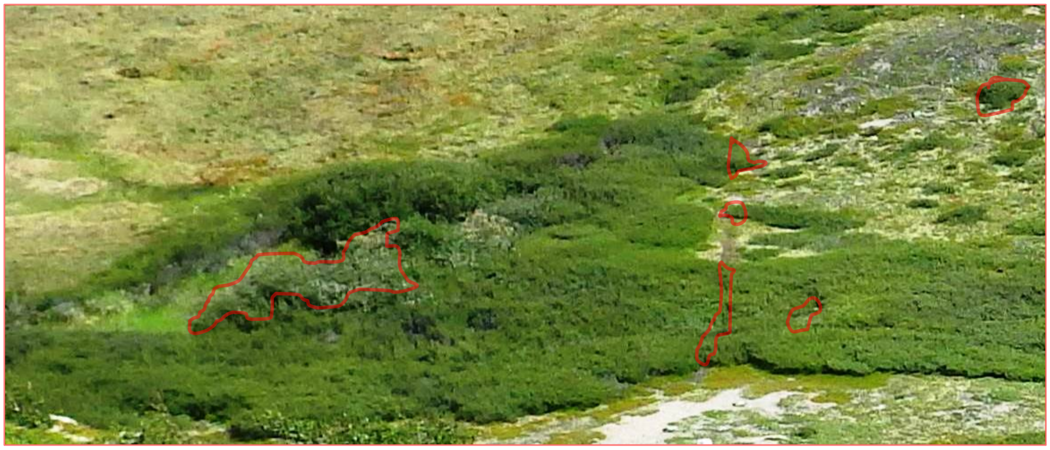

Esker Plain habitat-type:

July 9, 2004

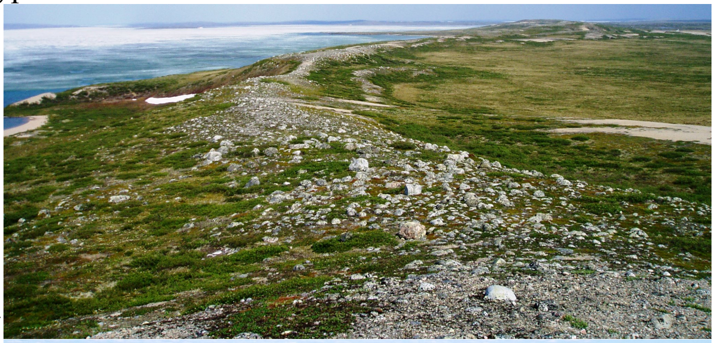

July 9, 2016

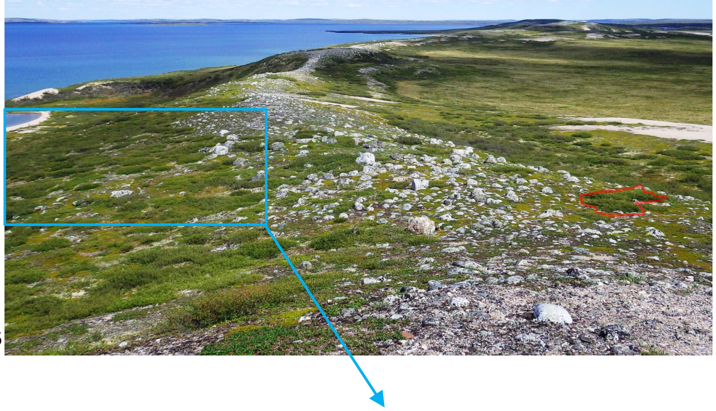

July 9, 2004

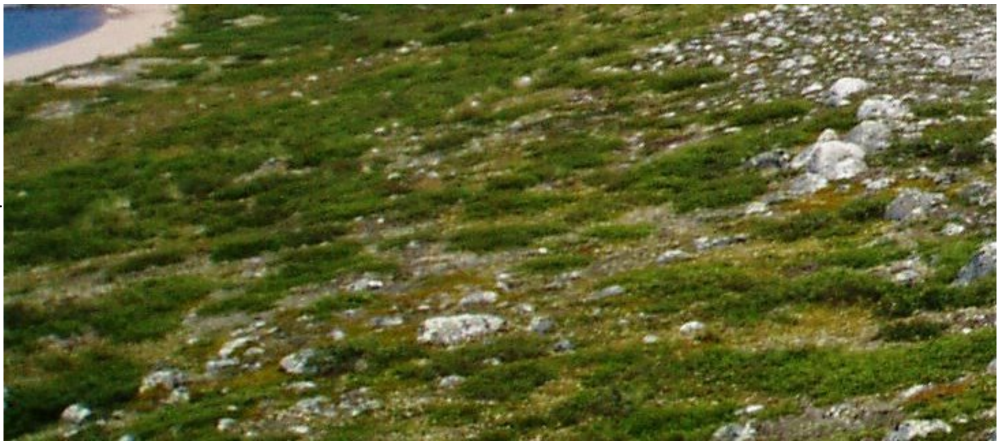

July 9, 2016

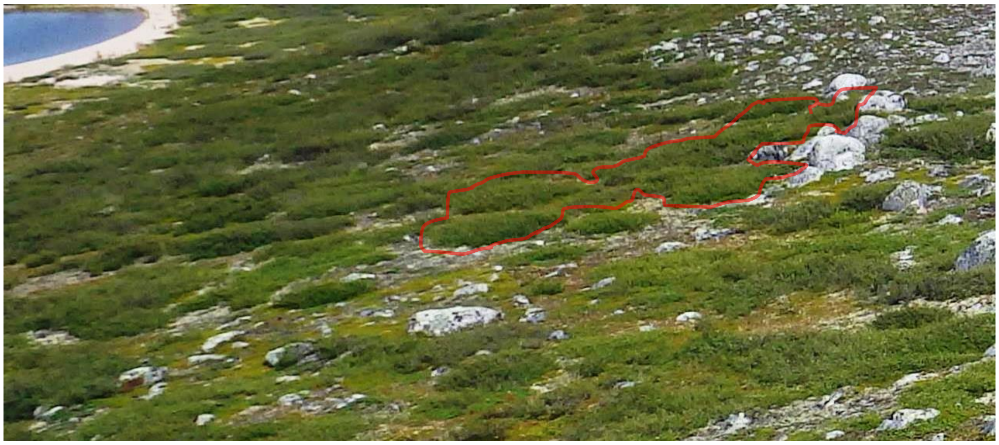

Multiple birch habitat-types including esker plain (top-slope), snowpack (mid- and lower slope), as well as tussock and sedge extending from the slope base across the foreground floodplain:

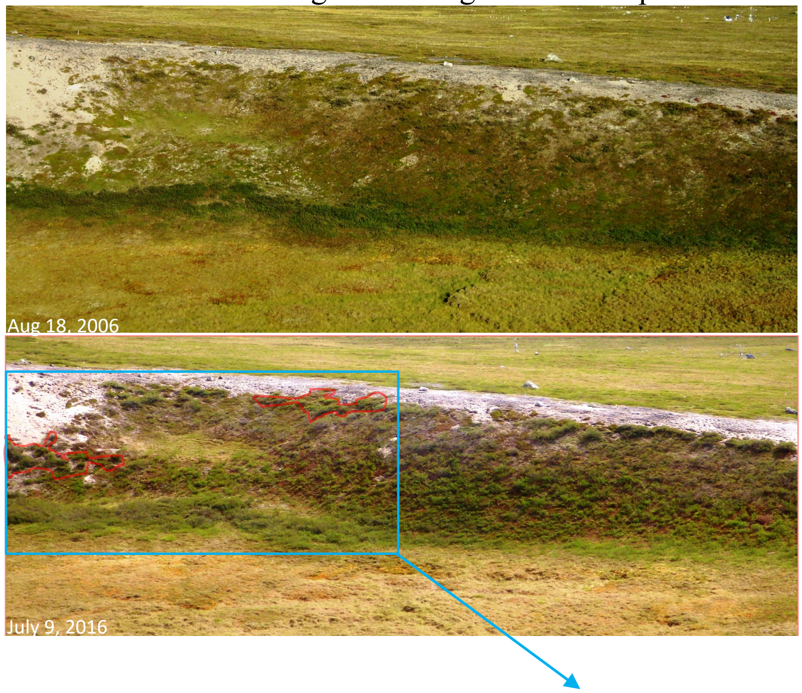

Aug 18, 2006

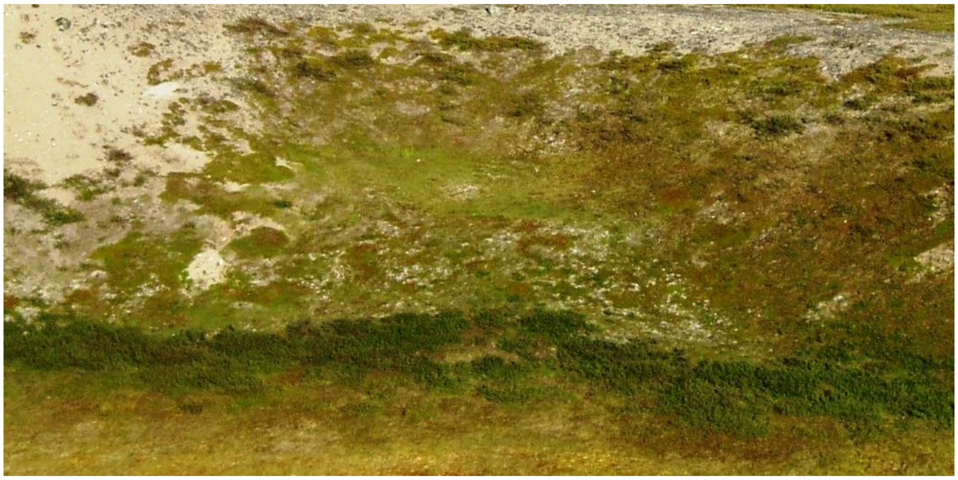

July 9, 2016

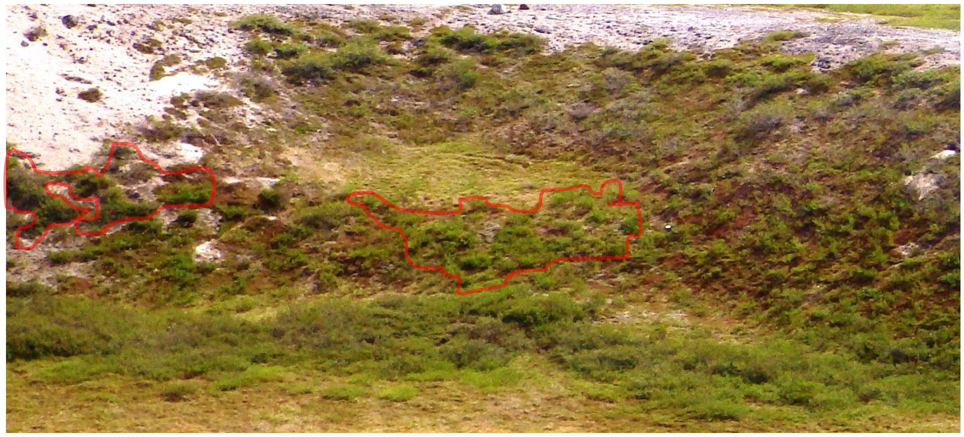

Appendix K. Specific measures taken by the authors to reduce the environmental impacts of the scientific activities reported in this study. The rationale for including this table is that every scientific lab and field activity involves environmentally-damaging impacts. As we become increasingly knowledgeable about the impacts that humanity's activities are having on the biosphere, we all – and scientists/researchers in particular – have a growing responsibility to acknowledge our impacts, and respond accordingly. Awareness is the fundamentally necessary pre-requisite to doing something about the problem. Inclusion of this table identifying and explicitly articulating specific measures that were deliberately taken in our science/research studies to minimize their environmental impacts is a first step toward advancing awareness. Note that the two measures included were probably the most environmentally significant, but even if not, the focus of this initiative at this stage is on the 'big picture' – awareness – not on the detail of comparing different measures or evaluating if the chosen measure was actually most effective in mitigating the impact.

**Category of science-associated activity**	**Details of the science-associated activity**	**Ecological impacts of the activity that may be avoidable**	**Measure taken to reduce those ecological impacts**
Travel	Travel from Queen's University in Kingston to Toronto airport for commercial flight to Yellowknife.	Greenhouse gas emissions associated with Kingston-Toronto flights	Travelled from Kingston-Toronto airport by train or bus instead of flying.
Travel	Chartered air travel from Yellowknife to the remote tundra research field-site at Daring lake each summer for various different science research groups including multiple universities and government scientists	Greenhouse gas emissions associated with each charter flight	Careful, ongoing, scheduling coordination of travel plans among the different research groups maximised the number of passengers per flight, and minimised the total number of flights
